# Nutritional Counseling During Chemotherapy Treatment: A Systematic Review of Feasibility, Safety, and Efficacy

**DOI:** 10.3390/curroncol32010003

**Published:** 2024-12-24

**Authors:** Shalet James, Alexie Oppermann, Kaitlin M. Schotz, Mackenzie M. Minotti, Gautam G. Rao, Ian R. Kleckner, Brenton J. Baguley, Amber S. Kleckner

**Affiliations:** 1 Department of Pain and Translational Symptom Science, University of Maryland School of Nursing, Baltimore, MD 21201, USA or sj1473@mynsu.nova.edu (S.J.); alexieoppermann@umaryland.edu (A.O.); ian.kleckner@umaryland.edu (I.R.K.); 2 Department of Psychology and Neuroscience, Nova Southeastern University, Fort Lauderdale, FL 33314, USA; 3 University of Maryland Medical System, Baltimore, MD 21201, USA; kaitlinschotz@umm.edu (K.M.S.); mackenzie.minotti@sluhn.org (M.M.M.); 4 University of Maryland Marlene and Stewart Greenebaum Comprehensive Cancer Center, Baltimore, MD 21201, USA; grao@umm.edu; 5 St. Luke’s University Health Network, Easton, PA 18045, USA; 6 Department of Obstetrics, Gynecology and Reproductive Sciences, University of Maryland School of Medicine, Baltimore, MD 21201, USA; 7 Institute for Physical Activity and Nutrition, Deakin University, Geelong, VIC 3220, Australia; b.baguley@deakin.edu.au

**Keywords:** dietetics, diet, chemotherapy, intervention, oral intake

## Abstract

Dietary interventions during chemotherapy hold promise for clinical and supportive care outcomes. We systematically investigated the feasibility, safety, and efficacy of nutritional counseling conducted during chemotherapy. Studies prospectively implemented nutrition counseling during chemotherapy. Articles were identified from three databases—EMBASE, Cochrane Library, and SCOPUS—from inception to 1 October 2024. Feasibility, safety, and efficacy of outcome data were extracted. Among 44 publications, 39 studies recruited 98 ± 80 participants (range 15–360); 38/39 (97%) were randomized controlled trials. One-third (31%) were among patients with breast cancer. Interventions were divided into individualized nutritional counseling (*n* = 21), nutrition counseling plus exercise (*n* = 13), and nutrient-specific dietary patterns (*n* = 10). Many had goals to achieve established nutrition guidelines. Feasibility was high based on attendance at counseling sessions, retention, and/or food log analysis. Overall, there were minimal adverse events related to the interventions. Many studies showed between-group differences favoring the intervention group for body weight (8/24, gain or loss, according to goals), nutritional status (8/9), quality of life (3/10 without and 6/9 with exercise), cancer-related fatigue (7/10), chemotherapy tolerance (6/11), and treatment responses (3/13). In conclusion, nutritional interventions were feasible and safe for patients undergoing chemotherapy and demonstrated preliminary efficacy to improve nutritional status, fatigue, chemotherapy tolerance, and other outcomes.

## 1. Introduction

There are approximately 20 million new cancer cases every year worldwide [1]. Treatments for cancer and their side effects can significantly impact a person’s quality of life. Fatigue, pain, and other treatment-induced side effects and toxicities lead to dose-reductions of life-saving treatment as well as persistent symptoms that limit daily activities and reduce overall well-being for years after treatment [2].

Dietary interventions may help manage and reduce the side effects of chemotherapy, improve overall quality of life during treatment, and improve tumor response to treatment [3,4]. Furthermore, by making informed choices about their nutrition, patients can actively participate in their cancer care and experience a greater sense of independence [5,6]. Guidelines on nutrition for cancer patients include *nutrient quantity* and *diet composition* [3,7,8]. In regard to *quantity*, clinical practice tends to focus on adequate calorie and protein intake to prevent muscle loss, sarcopenia, and malnutrition, including oral nutrition supplements if nutrient intake is not adequate from food alone [3,7,9]. In regard to *dietary composition*, guidelines do not recommend specific dietary patterns but encourage a broad range of tolerable foods to meet nutrient needs while discouraging dietary supplements because of their potential to interfere with chemotherapy efficacy [3,8,9,10]. Guidelines also lack more specific recommendations that target specific clinical and supportive care outcomes during chemotherapy. Further, they lack guidance on *nutrient timing* (e.g., intermittent fasting), a more recent discussion in the nutrition field [11,12].

Translational nutrition research is beginning to show that specific dietary patterns have large effects on tumor development. For example, work from Augenlicht et al. has shown that Western-style diets, which are replete in macro- and micronutrients, promote tumor growth in rodent models [13,14]. In addition, work by Yee et al. showed that omega-3 fatty acid consumption influences breast tissue composition in humans [15,16]. Thus, dietary patterns that are more specific than current recommendations have potential to influence clinical and supportive care outcomes.

Herein, to guide the development of more specific dietary recommendations for various target outcomes, we sought to gain a better understanding of the nutritional interventions that have been tested during chemotherapy treatment. Establishment of feasibility and efficacy for dietary interventions for specific outcomes—weight management, quality of life, etc.—can enhance our understanding of the role of nutrition in cancer care, refine clinical practices, and support the development of targeted interventions that optimize patient outcomes and well-being. The aim of this systematic review is to evaluate the feasibility, safety, and efficacy of dietary interventions on health outcomes in patients undergoing active chemotherapy treatment for cancer.

## 2. Methods

This systematic review was performed in accordance with the Preferred Reporting Items for Systematic Reviews and Meta-Analysis (PRISMA) reporting guidelines [17] and the protocol was pre-registered in the PROSPERO database (CRD42023417079). While the pre-registered protocol describes all dietary interventions during chemotherapy and radiation, our preliminary search identified over 88 disparate articles. Thus, we narrowed the focus of this review to only studies among patients undergoing active chemotherapy and excluded dietary interventions that focused on therapeutic diets focused on influencing biomarkers of cancer progression (i.e., intermittent fasting and ketogenic diets). This approach is in accordance with the European Society for Clinical Nutrition and Metabolism guidelines for clinical practice in cancer patients [9]. An ethics approval was not necessary because we were not collecting new data from human participants.

### 2.1. Eligibility Criteria

Studies were eligible if they matched the following inclusion criteria as specified by population, intervention, study design, and outcomes: (a) population: human participants of any age with any cancer diagnosis undergoing active chemotherapy treatment. (b) Intervention: any standardized dietary pattern that modulated nutrient composition or quantity (except ketogenic diet, which is a short-term therapeutic diet being tested to enhance antineoplastic activities of treatment, as well as other clinical indications). Studies involving enteral nutrition, dietary supplements, or functional foods were excluded. Interventions that also included physical activity components were included. (c) Study design: prospectively assigned randomized controlled trials, cross-over studies, or single-armed studies. (d) Outcomes: feasibility/adherence to an intervention, safety, and/or clinical outcomes as presented by the researchers. Only full-text articles published in peer-reviewed journals were included; articles were excluded if they were not published in English, conference abstracts or posters, theses or dissertations, or protocols only.

### 2.2. Search Strategy

Systematic research was conducted using three databases—EMBASE, Cochrane Library, and SCOPUS—from inception to 1 October 2024. A combination of MeSH (Medical Subject Headings) terms and keywords related to nutritional interventions during chemotherapy was consistently applied for each database search entry (Table S1). Search results from all three databases were imported into EndNote (Clarivate, Philadelphia, PA, USA) and scanned for duplicates. Screening of the titles and abstracts was carried out collaboratively by SJ and AO and independently by AK, followed by a full-text review. Discrepancies in eligibility for full-text articles were resolved by all authors responsible for screening (SJ, AO, and AK). The reference lists of identified papers and relevant review articles were manually checked for additional studies fulfilling the inclusion criteria.

### 2.3. Data Extraction and Risk of Bias Assessment

The data extraction procedure followed the PRISMA statement [17]. SJ and AK independently extracted the study design, year published, sample size, participants’ demographics (i.e., age, sex), cancer type and chemotherapy treatment regimen, intervention type and frequency of intervention, feasibility in the form of adherence and/or retention, safety in the form of adverse events, and health outcomes as reported in the published studies. Any discrepancies were resolved with discussion. We inductively decided which outcomes to report in the results section; we reported outcomes with the highest frequency among all studies. Two reviewers (SJ and AK) independently assessed risk of bias using Cochrane Risk of Bias 2.0 for randomized controlled trials [18], identifying “high risk,” “some concerns,” and “low risk” of bias for each clinical trial, and differences were resolved with discussion (Figure S1).

## 3. Results

### 3.1. Study Design

**Figure 1:**
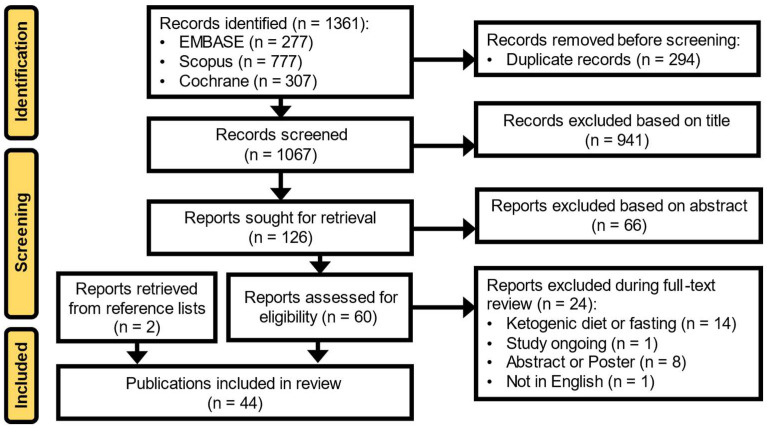
Preferred Reporting Items for Systematic Reviews and Meta-Analyses (PRISMA) flow diagram.

The systematic search identified 1361 articles from the EMBASE, Cochrane Library, and SCOPUS databases (Figure 1). After the removal of duplicates, evaluation of each article, and review of reference lists from the identified articles and relevant review articles, 44 publications met the full inclusion criteria and were included in this review [6,19,20,21,22,23,24,25,26,27,28,29,30,31,32,33,34,35,36,37,38,39,40,41,42,43,44,45,46,47,48,49,50,51,52,53,54,55,56,57,58,59,60,61].

### 3.2. Study Characteristics

The included studies were Phase I and Phase II clinical trials; there were no Phase III trials (Table 1) [6,19,20,21,22,23,24,25,26,27,28,29,30,31,32,33,34,35,36,37,38,39,40,41,42,43,44,45,46,47,48,49,50,51,52,53,54,55,56,57,58,59,60,61]. From the 44 publications, there were 39 trials because some of the studies resulted in more than one manuscript (i.e., Abdollahi et al. [19,28], Bourdel-Marchasson et al. [21,31], Sanft et al. [45,61], Kenkhuis et al. [59,60], and Kleckner et al. [6,52]). On average, the nutritional intervention studies recruited 98 participants, with a range of 15 (Maurer et al. [43])–360 (Jacot et al. [42]) individuals. Publications arose from studies conducted all over the world, including the United States (*n* = 9) [6,27,38,40,41,45,48,52,61], China (*n* = 7) [22,26,34,36,55,56,58], the Netherlands (*n* = 6) [33,46,49,50,59,60], France (*n* = 4) [21,31,39,42], Iran (*n* = 3) [19,28,51], the United Kingdom (*n* = 3) [20,35,37], Germany (*n* = 2) [29,43], Brazil (*n* = 2) [23,24], Denmark (*n* = 2) [30,47], India (*n* = 2) [44,53], Italy (*n* = 2) [54], Canada (*n* = 1) [57], Korea (*n* = 1) [25], and Thailand (*n* = 1) [32]. The most common patient population was breast cancer (*n* = 12/39, 31%) [19,23,24,27,38,39,40,41,42,45,53,54], followed by mixed cancer types (*n* = 9) [20,21,26,30,32,35,49,50,52]; hematologic cancers (*n* = 4) [29,47,48,51]; head, neck, and/or gastric cancer (*n* = 4) [22,34,37,58]; gynecological cancers (*n* = 3) [43,46,60]; colorectal cancer (*n* = 3) [33,44,57]; pancreatic cancer (*n* = 1) [25]; and lung cancer (*n* = 1) [55]. Six studies recruited patients undergoing concurrent [22,34,35,58] or sequential [39,42] radiation. Only five studies were published before 2010 (i.e., Ollenschläger et al., 1992 [29], Ovensen et al., 1993 [30], Loprinzi et al., 1996 [27], Gardner et al., 2008 [48], and Demark-Wahnefried et al., 2008 [40]).

### 3.3. Nutritional Interventions

The types of nutritional interventions varied greatly among studies (Table 1). More than half of the 39 total trials examined individualized nutritional counseling (*n* = 30), 19 without [19,20,21,22,23,24,25,26,27,29,30,32,33,34,35,36,56,57,58] and 11 with [37,38,39,40,41,42,43,44,45,46,60] added exercise regimens. All interventions were administered by a registered dietitian, trained coordinator, or unlicensed nutritionist throughout a patient’s cancer treatment; however, the interventions differed in dietary goals and format of counseling (e.g., face-to-face, telephone, video call, or a combination). These studies tended to set recommendations for minimum protein (~0.8–2.0 g/kg body weight) and calorie (~25–50 kcal/kg body weight) intake and/or encourage adherence to published guidelines, such as the European Society for Parenteral and Enteral Nutrition (ESPEN) [23] or the World Cancer Research Foundation (WCRF) [39]. Nine trials examined dietary patterns, including a Mediterranean diet (or a variation, *n* = 3) [51,52,54], a protein-rich home delivery program (*n* = 2) [49,50], high energy density [47], a neutropenic diet [48], a plant-based high-protein diet [53], or an anti-inflammatory diet [55].

### 3.4. Feasibility

There was not a standard manner in which studies reported feasibility. For example, some studies measured the percentage of counseling sessions attended (e.g., Bourdel-Marchasson et al. [21]), the number of participants who provided data at all time points (e.g., Keum et al. [25]), number of servings/amount of nutrients consumed from food logs (e.g., Ovesen et al. [30], Gardner et al. [48]), score on a diet composition-related questionnaire (e.g., Kleckner et al. [52]), or serum-based biomarkers (e.g., carotenoids, Djuric et al. [41]). Nutritional counseling resulted in high adherence among the 39 interventions in regard to number of sessions attended and/or compliance with goals, depending on what was reported (Table 2). Retention in the nutritional counseling trials was the lowest for one program with energy and protein goals among patients with esophageal cancer undergoing chemoradiotherapy [34] (67.9%); all others had ≥80% [22,24,25,30,57] or 90% retention [19,20,21,23,26,27,29,32,33,35,36,56,58]. Interventions incorporating exercise in addition to nutritional counseling reported slightly less adherence than nutritional counseling alone, and adherence to the exercise components was lower than to dietary counseling sessions. Retention for these studies was 70–<80% for four studies [38,41,43,60], 80–<90% for two studies [42,46], and ≥90% for six studies [37,39,40,44,45,55]. Interventions that included counseling sessions [38,40,45,46] reported high attendance (≥80% [38,45,46,60]). In regard to adhering to dietary recommendations, all [19,21,22,28,29,30,31,32,36,40,41,43,45,55,56,57,61] but one [60] of the 18 studies that reported these data demonstrated that participants changed their diets in the direction of the recommendations even if total adherence was not achieved; Kenkhuis et al. [60] studied a nutrition and exercise intervention and they noted no within-group or between-group changes in diet quality. Interventions tended to lead to higher calorie and/or protein intake compared to the control group, consistent with goals [21,22,30,32,36,43,50,57], or a decreased energy intake, consistent with goals [29]. Participants also increased fruit and vegetable intake in some [40,41,45,55] but not all [61] studies over time in the intervention group, consistent with recommendations, compared to the control group. Those that tested a Mediterranean diet during treatment saw a within-group increase in Mediterranean diet adherence score or similar measure in the intervention arm and not the control arm [43,52,54].

The trials that tested dietary patterns had high feasibility on average. Retention was >90% in six [48,50,51,52,53,54] of eight [47,48,49,50,51,52,53,54] trials, though in Bille et al. [47] there was a high number of dropouts between consent and initiation of the study; retention was 100% for participants who began the intervention. Studies that utilized a home delivery service to facilitate adherence reported that food provision was indeed a facilitator to adherence [6,49]; in IJmker-Hemink et al. [49] participants reported a median burden level of 2 on a scale from 0 to 10 [49].

### 3.5. Safety

Adverse events were reported in most studies, but events were primarily attributed to chemotherapy treatment rather than the intervention itself (Table 2). Nutritional counseling interventions appeared safe, with some studies indicating a lower incidence of chemotherapy-related side effects in the intervention group compared to the control [19,23,24,28,59,63]. Most studies reported no significant differences in side effects between intervention and control groups.

### 3.6. Primary Health Outcomes Assessed

The primary outcomes of the nutritional intervention clinical trials included feasibility as well as a variety of clinical and supportive care outcomes (Table 1). As many of these studies were Phase I or proof-of-concept studies, many declared the primary outcome measure to be feasibility (*n* = 7) [35,38,40,43,52,57,64]; Puklin et al. [61] specifically described changes in physical activity and diet quality attributable to the intervention. Common primary supportive care outcomes (or primary purpose of the manuscript) included quality of life (*n* = 10) [24,25,29,30,31,33,37,38,49,50], body mass and composition (*n* = 15) [22,26,27,30,32,36,38,40,43,46,47,54,57,60], cancer-related fatigue (*n* = 5) [39,42,52,53,55], and nutritional status (*n* = 5) [25,34,36,51,58]. The following primary outcomes were reported by a single study each: clinically relevant decrease in skeletal muscle area [33], anaerobic threshold measured by cardiopulmonary exercise testing (CPET) [37], chemotherapy-induced nausea and vomiting (CINV) [28], gastrointestinal side effects [19], infection [48], one-year survival [21], mortality [20], and relative dose intensity [45].

### 3.7. Efficacy

The most common clinical outcomes were body weight/body mass index (BMI), nutritional status, quality of life, cancer-related fatigue, chemotherapy toxicity, and chemotherapy responses/survival. These outcomes are summarized here.

#### 3.7.1. Body Weight or Body Composition

Body weight and/or BMI was reported by 25 studies [20,22,23,25,26,27,29,30,32,33,36,38,39,40,41,42,45,47,48,51,52,53,54,58,60]. Nine studies saw no between-group differences in changes in body weight/BMI [20,23,30,32,38,42,45,48,52]. Body weight was higher in the intervention vs. control group for nine studies (weight gain or attenuation in weight loss), consistent with goals [22,25,26,27,33,36,51,58,60] (for the LAM-6 remission induction chemotherapy regimen only in Ollenschläger et al. [29] and for the intervention vs. control periods for Bille et al. [47]). There were four trials that aimed to reduce body weight, specifically fat mass, during chemotherapy, and all showed a significant between-group reduction in weight [39,41,53,54]. Demark-Wahnefried et al. [40] noted no significant lean body mass losses in any group over time and no between-group differences, except the diet plus exercise arm, which had a decrease in waist circumference over time. Ford et al. [57] did not report body weight but observed that a greater protein intake was associated with increased muscle mass (as measured using appendicular lean soft tissue index).

#### 3.7.2. Nutritional Status

Nutritional status was measured in nine studies [22,25,26,32,34,36,51,55,58]. Studies monitored nutrition status using albumin levels [22,26,34,36,51,55], the Patient-Generated Subjective Global Assessment (PG-SGA) [25,32,55], and/or the Nutritional Risk Score [58]. Higher nutritional indexes were seen in the intervention vs. control group in eight [22,26,32,34,36,51,55,58] of nine [22,25,26,32,34,36,51,55,58] studies; Keum et al. [25] reported no between-group differences but observed that participants who used the Noom smartphone application experienced greater improvements in nutritional status.

#### 3.7.3. Quality of Life

Quality of life was measured using the European Organization for Research and Treatment of Cancer (EORTC) Quality of Life Questionnaire (QLQ)-C30 [20,24,25,28,31,33,37,42,43,50,55], a Functional Assessment of Chronic Illness Therapy (FACIT) questionnaire [20,40,41,52], the 36-item Short Form Survey [58], a single-item question [29], the Concise Quality of Life Index [30], the Thai-Modified Function Living Index Cancer Questionnaire [32], the 5-level EuroQual (EQ-5D-5L) questionnaire [35], or the Functional Living Index-Emesis [56] (Table 2). Ten [20,24,25,29,30,31,33,35,56,58] of 11 [20,24,25,29,30,31,33,35,56,58] nutritional counseling studies without added exercise saw no differences between groups for quality of life. Najafi et al. [28] reported higher quality of life in the intervention group, though both groups had stable scores over time, and Sukaraphat et al. [32] reported greater quality of life in the intervention vs. the control group post-intervention. The exercise-plus-nutrition interventions were associated with higher quality of life vs. the control group for six [37,38,39,41,43,55] of nine [37,38,39,40,41,42,43,55,60] studies. Both the high protein [50] and the Mediterranean diet [52] interventions led to greater quality of life vs. the control, but the difference did not reach statistical significance.

#### 3.7.4. Cancer-Related Fatigue

Cancer-related fatigue was measured in eight studies [28,39,42,43,52,53,55,60]. These trials used the EORTC QLQ-C30 fatigue scale [28], Multidimensional Fatigue Inventory [39,42,43,60], the Chinese version of the Brief Fatigue Inventory [55], the FACIT-F fatigue subscale [52], or the Fatigue Symptom Inventory [53]. Improvements in fatigue were observed in the intervention vs. control group for six studies [28,39,43,52,53,55] and were similar between groups in two studies of nutrition plus exercise [42,60].

#### 3.7.5. Chemotherapy Tolerance

Eleven studies [21,23,26,32,35,36,37,39,45,53,59] reported tolerability to chemotherapy by means of relative dose intensity or chemotherapy completion rate [35,37,39,45,53,59], chemotherapy interruptions or postponements [21,26,32,36], or dose-limiting toxicities [23]. Despite the broad range of nutritional interventions, there were between-group differences favoring the intervention group or intervention period for six of the studies [23,26,32,35,36,37]; there were no between-group differences for five studies [21,39,45,53,59].

#### 3.7.6. Response to Chemotherapy

Tumor response and/or survival were reported by 13 publications [20,21,23,25,30,32,33,34,37,44,45,48,59]. These outcomes included tumor size or response to treatment [25,30,32,44,45,48], remission status [34,59], and survival or mortality statistics [20,21,23,25,33,37,59]. Between-group differences favoring the intervention group were seen in three studies—one encouraging adequate protein, calories, and micronutrients according to Dutch recommendations [33]; one encouraging macronutrients and American food group recommendations [45]; and one testing naturopathy, yoga, and a specific provided Indian diet [44]—with no between-group differences in 10 studies [20,21,23,25,30,32,34,37,48,59]. Keum et al. [25] observed a greater tumor response rate for “above average” Noom smartphone application users vs. non-Noom users.

#### 3.7.7. Other Outcomes

Several other outcomes were not commonly reported but were noteworthy. Abdollahi et al. [19], de Lima Bezzera et al. [23], and White et al. [35] targeted gastrointestinal side effects such as nausea, vomiting, and diarrhea, and notable improvements were observed for the nutritional counseling intervention vs. control group. Cao et al. [56] performed a nurse-led multidomain intervention among patients with head and neck cancers and saw significant reduction in the incidence of nausea and rate of vomiting attributable to the intervention. The neutropenic diet intervention, which assessed infection and mortality rates, revealed that the diet did not prevent major infections or death [48].

## 4. Discussion

The aim of this systematic review was to evaluate the feasibility, safety, and preliminary efficacy of dietary interventions in patients undergoing active chemotherapy treatment for cancer. The results suggest individualized nutritional counseling to meet current nutrition guidelines and/or mitigate chemotherapy-induced side effects can improve patient outcomes while being safe and feasible alone or when combined with exercise interventions. In addition, more specific dietary patterns, including an Indian naturopathy dietary pattern [44], the Mediterranean diet [51,52], and a plant-based high-protein diet [53], were feasible. Nutritional counseling and dietary pattern interventions tended to be effective for managing body weight during treatment, whether it was to (a) increase body weight or attenuate weight loss or (b) minimize increases in fat mass (Table 2). Nutritional counseling interventions consistently improved nutritional status and nutritional indexes. In regard to quality of life, interventions were more effective if exercise was combined with nutrition counseling. Three-quarters (6/8) of nutritional counseling or specific dietary patterns improved cancer-related fatigue. In regard to clinical outcomes, there were between-group differences favoring the intervention group or intervention period for approximately half (6/11) of the studies assessing tolerance to chemotherapy over a broad range of nutritional interventions, and 3/13 interventions favored the intervention group in regard to tumor response and/or survival metrics, with the others showing no between-group differences. None of these trials were Phase III definitive trials, and most of these efficacy measures were not primary outcomes, though these data provide support for future trials testing nutritional interventions during chemotherapy treatment.

The Academy of Nutrition and Dietetics [65], the European Society of Enteral and Parenteral Nutrition (ESPEN) [3,9], and the American Cancer Society [7] publish nutrition guidelines for patients with cancer undergoing chemotherapy, with less formal guidelines for the geriatric oncology population [66,67]. These guidelines aim to detect, treat, and prevent malnutrition, specifically with 25–30 kcal/kg body weight/day and 1.0–1.5 g protein/kg body weight/day with micronutrient needs, in accordance with the Recommended Dietary Allowance (RDA) for age [3,7,9]. The Mediterranean diet, a high-protein plant-based diet, and an anti-inflammatory diet support these guidelines and can be tailored to a patient’s preferences (e.g., flavors, protein choices), circumstances (e.g., socioeconomic status, social patterns), and clinical needs. The wide range of energy and protein recommendations among our included studies (e.g., 0.8–1.2 g protein/kg body weight in Sukaraphat et al. [32] and 2.0 g/kg in Ford et al. [57]) may have resulted in the differences in efficacy for some of the reported outcomes, such as changes in body weight.

A total of 28 trials tested individualized nutrition counseling programs to promote adherence to established guidelines while mitigating treatment-related side effects and toxicities (Table 1). These studies imply that, despite the existence of general guidelines, directed, personalized education, interpretation, and/or implementation is needed from a dietitian to achieve adherence. This is likely because patients have diverse nutrition needs based on cancer type, pre-treatment body composition, menopausal status, barriers to consuming food, etc. For example, cancers such as head and neck and colorectal cancers more directly interfere with the ability to consume and absorb nutrients; these patients may require modified texture or food types. Some types of cancers are associated with weight gain (e.g., breast cancer) [68] and some are associated with weight loss (e.g., pancreatic cancer), and therefore dietitians may have different priorities for energy needs [3,9]. Furthermore, different symptom profiles such as nausea, mucositis, and diarrhea require individualized nutrition strategies to meet nutrient goals. As nutrition recommendations become more specific, interventions may become contraindicated or require modifications.

This systematic review highlights gaps in the literature. Nutrition in oncology as a field is beginning to move beyond macronutrient recommendations to more specific nutrient recommendations and dietary patterns. More research into the Mediterranean diet [51,52,54], plant-based high-protein diets [53], anti-inflammatory diets [55], and other diets can further guide individualized, structured programs for patients. Overweight status is highly prevalent at large as well as in the oncology population [69]. Studies specifically examining relationships between BMI at presentation and controlled weight change through treatment are needed. In addition, approximately half of patients with cancer are older adults, some of whom have different metabolic profiles (e.g., decreased anabolic response to protein) and goals of treatment (e.g., minimize dose reductions, maintain independence [70]); nutrition interventions should be tested specifically in older adults with relevant physical and functional outcomes [66]. More research should explore the integration of digital platforms to support dietary monitoring and adherence (such as the smartphone app in Keum et al. [25] and a web-based program in Chan et al. [71] recruiting men undergoing chemotherapy or radiation). Ketogenic diets and intermittent fasting regimens were beyond the scope of this review but are an active area of research, mostly as a short-term intervention to improve clinical outcomes [12,72,73,74]. While whole-food plant-based diet tends to meet society guidelines for optimizing nutrition and reducing future chronic disease risk, only a few studies have begun to rigorously test the effects (e.g., Campbell et al. [75]).

There is a plethora of mechanisms by which nutritional interventions can improve outcomes, many of which are active areas of research. First, nutritional counseling and many dietary patterns confer biological and metabolic benefits such as correcting nutritional deficiencies [25,32,55], reducing chronic inflammation [34,55], improving mitochondrial energy metabolism [52], and combating oxidative stress [76]. Indeed, switching from a Western diet to a healthy diet after colorectal cancer initiation improved colon health and symptoms in a mouse model [77]. Future research should look into the ability of dietary interventions during chemotherapy to improve the health of the gut microbiome [78] and regulate circadian rhythms [11]. Second, nutritional interventions can confer psychological benefits, including increases in self-efficacy and mood [79], as well as expectancy effects [80]. Third, dietary interventions can result in social benefits by bringing people together, whether it is with friends, family, the clinical care team, or peers in support groups [81,82].

Sex differences in cancer involve variations in the incidence, survival rates, and treatment response between males and females across various cancer types [83,84]. Research suggests that genetic, hormonal, and biological factors contribute to these differences [85]. Sex introduces variations in dietary outcomes attributed to metabolic differences, as seen in animal studies [86], as well as in organ-specific and hormone-related cancers. We noted a scarcity of research that systematically investigates how these interventions affect males and females differently. Understanding how dietary changes influence cancer outcomes in a sex-specific manner could have profound implications for lifestyle behaviors, treatment strategies, and cancer survivorship.

This work has important clinical implications. There is now clear evidence that individual dietary counseling to meet calorie and protein recommendations can mitigate side effects, maintain weight/fat-free mass, maximize tolerated dose, prevent treatment delays, mitigate reductions in performance status, and improve quality of life during treatment (Table 2) [3,7]. While ensuring that minimum protein and calorie needs are being met should remain the first priority to prevent malnutrition [3,7,9,65], many patients may be in a position to adopt more detailed dietary patterns in regard to macro- and micronutrients, food groups, and nutrient timing. Unfortunately, many cancer treatment centers have inadequate availability of licensed nutrition practitioners [87,88,89]. A clinical workflow that includes dietitians in outpatient cancer care for all patients undergoing chemotherapy, with tools to self-promote adherence, is needed to improve implementation of guidelines and leverage the benefits of optimizing nutrition throughout cancer care.

This review has both strengths and limitations. First, we compiled all interventions conducted during chemotherapy treatment, gathering an all-encompassing understanding of the current state of nutritional interventions in this population. The risk of bias for these studies tended to be low (Figure S1), with potential risks being not preregistering the study (especially for older studies) and incomplete description of the randomization process. One limitation of this systematic review is that the interventions, control groups, and outcomes (types and methods of measuring them) exhibited considerable heterogeneity, precluding the ability to conduct a useful meta-analysis. Meta-analyses are instrumental in generating a composite effect size for an intervention in comparison to a control group [17]. However, given the diverse nature of the studies included in this review, combining the data in a meta-analysis would not yield meaningful insights to guide further intervention development or inform clinical implementation. Second, adherence to interventions was not standardized, and therefore it was not possible to quantitatively assess the relative feasibility of interventions. However, we were able to qualitatively assess feasibility. Third, each cancer type, stage, and process has wildly different implications for baseline patient characteristics and needs, and there are different side effects of the disease process itself. Dietitians individualize recommendations based on these factors in addition to chemotherapy-related side effects and needs. Due to the limited number of studies that tested the same intervention within the same populations, we are not yet at the point where we can make more specific recommendations based on disease type, age, sex, etc. Fourth, our review did not include studies testing oral nutritional supplements, which are common and efficacious for many patients [90]. Finally, some publications that tested nutritional interventions during cancer treatment were not eligible for our review because they recruited patients undergoing either chemotherapy or radiation treatment (e.g., Movahed et al. [91], Chan et al. [71]). However, by excluding these we are able to report results for only patients undergoing active chemotherapy.

## 5. Conclusions

In conclusion, this systematic review shows that dietary interventions that include or go beyond current nutritional guidelines during chemotherapy treatment tend to be feasible, safe, and effective for a multitude of clinical and supportive care outcomes. Further, more definitive research is necessary to establish an evidence base that is strong enough for inclusion in guidelines and incorporation into the clinical infrastructure to improve the outcomes for patients undergoing chemotherapy.

## Figures and Tables

**Table 1:** Characteristics of the studies included in this systematic review.

Reference (Country)	Study Design	Sample Size	Eligibility	Intervention Type	Intervention Details	Primary Outcome(s) (Not Including Feasibility)
***Nutrition counseling and/or food provision without exercise***						
Abdollahi et al., 2019 (Iran) [19]	Randomized controlled trial	Randomized N = 150	Female adults (≥18 y) with breast cancer who have received one session of chemotherapy (any type) and have a history of constipation or diarrhea after chemotherapy.	Nutrition counseling	Trial duration: 10-week trial. Intervention: Face-to-face nutrition education consisting of a diet with **12–15% calories of protein, 30–35% of fat, and 55–60% of carbohydrates estimated** individually based on participant’s current age, weight, and height in addition to nutrition recommendations to reduce diarrhea and prevent reflux. Control: Control group had usual care without informational pamphlet, nutritional education, or dietary intervention, and they were asked not to change their usual diets.	Gastrointestinal side effects—nausea, vomiting, and diarrhea
Baldwin et al., 2011 (United Kingdom) [20]	Randomized controlled trial	Randomized N = 277	Adults with metastatic or locally advanced cancer of the gastrointestinal tract, lung, or mesothelioma on any type of chemotherapy; lost weight in the last 3 months; agreed to undergo and were fit for palliative chemotherapy	Nutrition counseling	Trial duration: 6 weeks, with a 52-week follow-up. Intervention 1: Advice to **increase food intake by 600 kcal** (2510 kJ) per day with the intent to increase body weight; booklet contained 150 kcal ideas and patients were asked to select 4/day. Intervention 2: A 588 kcal (2460 kJ) sachet of nutritional supplement to be prepared in whole milk + a daily multivitamin Control: No dietary intervention.	Survival
Bourdel- Marchasson et al., 2014 (France) [21]	Randomized controlled trial	Randomized N = 341	Patients older than 70 y with lymphoma or carcinoma with an indication of chemotherapy (any type); participants needed to be at risk of malnutrition with a full Mini Nutritional Assessment: 17–23.5 points	Nutrition counseling	Trial duration: 3 to 6 months, each participant received 6 visits. Intervention: Aim of achieving an energy intake **of 30 kcal/kg body weight/d and 1.2 g protein/kg/d** by face-to-face discussion targeting the main nutritional symptoms, compared to usual care. Control: Usual nutritional care given in cancer treatment settings. There were no restrictions for dietary advice or oral supplements.	One-year survival
Cao et al., 2024 (China) [56]	Randomized controlled trial	Randomized N = 92	Adults (≥18 y) diagnosed with head and neck squamous cell carcinoma, planned to receive first round of chemotherapy (cisplatin-based)	Nutrition counseling	Trial duration: 24 weeks. Intervention: Nurse-led multidomain intervention of chemotherapy-induced nausea and vomiting (CINV) included nurse-led risk assessment, education on prevention and control of CINV, antiemetic treatments following guidelines, dietary strategies (based on the National Comprehensive Cancer Network), relaxation therapy, and follow-up. Individualized dietary counseling was provided by the nurse tailored to patients’ specific needs. Control: Usual care of CINV consisting of administration of antiemetics, education about CINV control, and dietary recommendations provided by primary nurses.	Incidence of CINV, severity of CINV, influence of CINV on patients’ quality of life
Dai et al., 2022 (China) [22]	Pilot randomized controlled trial	Randomized N = 72	Men and women aged 18–75 y, head or neck cancer, concurrent chemoradiation therapy (nedaplatin)	Nutrition counseling	Trial duration: Duration of the concurrent chemoradiation therapy. Intervention: Face-to-face nutritional counseling at least once every 2 weeks during treatment to achieve **calorie and protein requirements** determined by the ESPEN guidelines on nutrition in cancer patients. Control: Nutrition treatment plans according to clinical experience.	Body weight
de Lima Bezerra et al., 2023 (Brazil) [23]	Randomized controlled trial	Randomized N = 34	Adult women with breast cancer, indicated for neoadjuvant chemotherapy (any type)	Nutrition counseling	Trial duration: 2 months. Intervention: Personalized diet with an **individually tailored meal plan based on ESPEN guidelines** to address malnutrition [9] (25–30 kcal/kg body weight/day and 1.5 g protein/kg/day). Control: Counseling on healthy eating and nutritional information on how to reduce the severity of chemotherapy-induced nausea and vomiting.	Chemotherapy toxicity
de Souza et al., 2021 (Brazil) [24]	Randomized controlled trial	Randomized N = 34	Female adults with breast cancer indicated for neoadjuvant chemotherapy (doxorubicin and cyclophosphamide)	Nutrition counseling	Trial duration: 3 cycles of chemotherapy (21 days/cycle). Intervention: A personalized diet meal plan formulated by a dietitian based on age, current weight, and height of each subject, **25–30 kcal/kg/day of energy and 1.5 g/kg/day of protein, based on the ESPEN guidelines** [9], including written materials on healthy eating and symptom management Control: Pamphlets with recommendations on healthy eating and to reduce chemotherapy-induced nausea and vomiting.	Quality of life
Ford et al., 2024 (Canada) [57]	Randomized controlled pilot trial	Randomized N = 50	Adults (18–85 years) with stage II–IV colorectal cancer starting any type of chemotherapy	Nutrition counseling	Trial duration: 12 weeks. Intervention groups: Weekly telephone nutrition education with patients to achieve **2.0 g protein/kg body weight/day** and meal patterns consistent with those recommended by the Academy of Nutrition and Dietetics. Control: Weekly telephone nutrition education with patients to achieve a protein intake of 1.0 g/kg/day (standard of care). Both: Prescribed diets were translated into an individualized daily meal pattern based on typical dietary intake.	Muscle mass and physical function
Keum et al., 2021 (Korea) [25]	Randomized controlled trial	Randomized N = 40	Males or females aged 20–70 y with pancreatic ductal adenocarcinoma scheduled to receive chemotherapy (any type)	Nutrition counseling via a smartphone app	Trial duration: 12 weeks. Intervention: **Noom** is a mobile app that offers a curriculum and human coaching. It was tailored to the population and included food logging, feedback-based food choices, daily articles for health, and coach–participant messaging with goals **to encourage calorie intake and maintain nutritional status**. Control: Usual care.	Nutritional status and quality of life
Lin et al., 2017 (China) [26]	Randomized controlled trial	Randomized N = 110	Males and females aged ≥18 y with cancer (any type) and undergoing chemotherapy (any type)	Nutrition counseling	Trial duration: 12 cycles of chemotherapy (duration in days not specified). Intervention: **Individual recipes based on estimates of total energy expenditure** to achieve or maintain high nutritional status. Participants and their caretakers were counseled by experienced nurses, and diets were adjusted as nutritional status changed throughout the intervention. Control: Usual care plus nutrition screening, nutritional guidance, diet mission, and arranged mealtimes.	Patient weight and serum albumin and prealbumin levels
Loprinzi et al., 1996 (United States) [27]	Randomized controlled trial	Randomized N = 109	Premenopausal women with resected localized breast cancer scheduled to receive adjuvant chemotherapy (any type)	Nutrition counseling	Trial duration: Duration of chemotherapy, assessments at 3 and 6 months after initiation of chemotherapy. Intervention: Counseling with a dietitian prior to or within 2 weeks of chemotherapy initiation and at 4-to 6-week intervals. Dietitian goals included **maintaining on-study body weight or gradual weight reduction toward ideal body weight**. Control: Standard care—information on the potential for weight gain and ways to prevent it.	Body weight
Najafi et al., 2019 (Iran) [28]	Same as Abdollahi et al. [19]	Randomized N = 150	Females aged 18–60 y with stage IA–IIIB breast cancer being treated with thrice-weekly cycles of doxorubicin, cyclophosphamide, and docetaxel and had experienced vomiting during or after the previous session(s) of chemotherapy	Nutrition counseling	Trial duration: Duration of chemotherapy. Intervention: Personalized diet and nutrition education before each chemotherapy session. The daily diet was estimated individually and contained **1.2–1.5 g/kg of protein, 30% of energy from fat, and 55–60% of energy from carbohydrates**. Control: Usual care.	Chemotherapy-induced nausea and vomiting
Ollenschläger et al. (Germany) 1992 [29]	Randomized controlled trial	Enrolled N = 32	Males and females aged 17–60 years with acute lymphocytic or nonlymphocytic leukemia; had unintentional weight loss of >5% in the previous 3 months and/or actual body weight below 90% ideal body weight; planned cytostatic treatment with lamivudine-6; thioguanine, Ara-C, and daunorubicin (TAD-9); or the Ulm protocol	Nutrition counseling	Trial duration: Duration of therapy from induction of therapy until planned or premature end of maintenance therapy (4–54 weeks); participants were hospitalized for some of their cancer treatment and some were on an out-patient basis. Intervention: “Intensified oral nutrition program”: ad libitum menu of **1.0–2.0 g protein and 30–50 kcal/kg body weight plus daily visit by a dietitian** to assess nutritional status, nutritional behavior, nutrient requirements, symptoms, and well-being. Control: Free choice, ad libitum menu of 1.0–2.0 g protein and 30–50 kcal/kg body weight.	Quality of life
Ovesen et al., 1993 (Denmark) [30]	Randomized controlled trial	Enrolled N = 137	Males and females with small-cell lung cancer, ovarian cancer, or breast cancer on any type of chemotherapy	Nutrition counseling	Trial duration: 5 months. Intervention: Counseling with a dietitian before chemotherapy and once–twice monthly and as needed; goals to meet the **protein and calorie requirements of the Nordic Recommended Allowances** (1.5–1.7 × basal energy expenditure as calculated by the Harris–Benedict Equation; 1.0–1.2 g protein/kg body weight) Control: Ad libitum diet. Both groups: Advised to supplement diet with one multivitamin tablet that contained the recommended daily allowances of micronutrients.	Body weight, quality of life
Regueme et al., 2021 (France) [31]	Same as Bourdel-Marchasson et al. [21]	Randomized N = 283				Health-related quality of life
Sukaraphat et al., 2016 (Thailand) [32]	Randomized controlled study	Enrolled N = 50	Adults (age ≥ 18 years) with locally advanced unresectable or metastatic cancer on first-line chemotherapy (any type)	Nutrition counseling	Trial duration: 9–12 weeks (3–4 cycles of chemotherapy). Intervention: Individualized and intensive dietary counseling based on the **ESPEN guideline** [3]: **30–35 kcal/kg body weight and 0.8–1.2 g protein/kg body weight per day** with regular food, supplements used if required. Control: Routine care: dietary counseling for general dietary recommendations from a nurse and physician but not a dietitian.	Body weight
Tang et al., 2024 (China) [58]	Quasi-experimental study	Randomized N = 60	Adults (≥18 y) with nasopharyngeal carcinoma who were on this type of chemotherapy (any type) and had been diagnosed with malnutrition (weight loss ≥ 5%) and had a life expectancy of more than 3 months.	Nutrition counseling	Trial duration: 24 weeks. Intervention: Face-to-face nutrition education with patients and their families consisting of a diet **high in protein, high in calories, high in vitamins, and rich in inorganic salts**, in addition to nutrition recommendations to avoid dry mouth, oropharyngeal pain, and constipation consistent with guidelines. Control: Routine dietary guidance without nutrition assessment screening.	Nutritional Risk Screening 2002, BMI, and quality of life
van der Werf et al., 2020 (Netherlands) [33]	Randomized controlled trial	Randomized N = 107	Males and females aged > 18 y diagnosed with metastatic colorectal cancer, scheduled for first-line chemotherapy with capecitabine and oxaliplatin (CAPOX), infusional 5- fluorouracil and oxaliplatin (FOLFOX) or capecitabine alone, with or without bevacizumab	Nutrition counseling	Trial duration: Duration of chemotherapy (varied by participant based on regimen). Intervention: Face-to-face counseling (at every chemotherapy infusion) and telephone consultation (between infusions) by a dietitian with the **goal of achieving sufficient protein, energy, and micronutrient intake. The protein intake goal was ≥1.2 g protein/kg body weight/day**. The dietitian also encouraged moderate-intensity physical activity for ≥30 min per day, five days per week. Control: Access to a dietitian.	Skeletal muscle area (primary), quality of life, physical functioning, treatment toxicity, treatment intensity, survival (secondary)
Wang et al., 2023 (China) [34]	Randomized control trial	Randomized N = 53	Males and females aged 50–80 years with esophageal cancer undergoing chemoradiotherapy (cisplatin + paclitaxel or cisplatin + 5-fluorouracil + radiation)	Nutrition counseling	Trial duration: Duration of chemoradiotherapy (5–8 weeks). Intervention: Individualized nutrition programs with **target energy and protein intake (30–35 kcal/kg body weight/day and 1.2–1.5 g protein/kg/day**). Nutritional evaluations were conducted by nutrition nurses each day, and nutritional interventions were adjusted based on needs. Control: Usual care.	Nutritional status
White et al., 2020 (United Kingdom) [35]	Randomized control trial	Randomized N = 50	Males and females aged > 18 y, with cervical or bladder cancer, undergoing chemotherapy and radical pelvic radiotherapy (weekly gemcitabine for bladder cancer or cisplatin for cervical cancer)	Nutrition counseling	Trial duration: Not specified, 6-week, 12-week, and 12-month follow-up. Intervention: Individualized dietary counseling using the anthropometric data, patient-reported subjective, Global Assessment score, Malnutrition Universal Screening Tool score, dietician symptom assessment, and 3-day food diary to tailor dietary advice and prescription of oral nutritional supplements where necessary. Control: Not stated.	Feasibility (primary), treatment toxicity (primary clinical outcome)
Xie et al., 2017 (China) [36]	Randomized control trial	Randomized N = 144	Males and females aged ≥ 18 y with gastric cancer undergoing post-operative chemotherapy (oxaliplatin and capecitabine every 21 days for up to 8 cycles)	Nutrition counseling	Trial duration: Duration of chemotherapy. Intervention: **Intensive individualized nutritional and educational interventions** during the entire course of chemotherapy. Control: Education on chemotherapy, possible chemotherapy-induced adverse events, basic nutrition guidance, follow-up 2 days after infusion.	Nutritional status
***Nutrition counseling plus exercise***						
Allen et al., 2022 (United Kingdom) [37]	Pilot randomized controlled trial	Randomized N = 54	Adults with locally advanced esophagogastric cancer planned for neoadjuvant therapy (any type) plus esophagogastrectomy, or total gastrectomy.	Nutrition counseling and exercise	Trial duration: 15-week trial (preoperative). Intervention: Needs-based, frequent, and tailored to the patient by dietitians to **increase total calories and protein** where appropriate. This group also received a structured exercise program. Control: Standard care, no nutrition or exercise prehabilitation.	Cardiopulmonary exercise test performance, muscle mass, and quality of life
Basen-Engquist et al., 2020 (United States) [38]	Pilot randomized controlled trial	Randomized N = 39	Patients with newly diagnosed stage II or III breast cancer within 3 weeks of starting neoadjuvant chemotherapy (any type)	Nutrition counseling and exercise	Trial duration: ~6 month trial (until the end of chemotherapy). Intervention: 20 individualized counseling sessions, 14 in person and 6 by telephone. Promoted foods with **low energy density** and consistent with recommendations for survivors. Also, an exercise intervention focused on resistance and flexibility training to maintain or increase fat-free mass. Control: Provision of publicly available written materials on cancer survivorship.	Anthropometrics (to prevent weight gain), quality of life, serum biomarkers
Brouwer et al., 2024 (Netherlands) [59]	Randomized controlled trial	Randomized N = 81	Patients aged > 18 years with primary epithelial ovarian cancer who were scheduled for (neo)-adjuvant chemotherapy treatment (six 3-week cycles of adjuvant carboplatin with or without paclitaxel for early FIGO stage and adjuvant or neo-adjuvant carboplatin or cisplatin and paclitaxel for advanced FIGO stage).	Nutrition counseling and exercise	Trial duration: From first or second chemotherapy cycle until 12 weeks after the last chemotherapy cycle. Intervention: Two 1 h sessions per week, supervised by a physical therapist specialized in oncology, including moderate- to **high-intensity resistance and aerobic exercise**. Dietary counseling was delivered by dietitians specialized in oncology once every 3 weeks during 30–45 min face-to-face sessions in the hospital or by telephone tailored to the nutritional needs of each patient. All participants were advised to follow the dietary guidelines set by the **World Cancer Research Fund (WCRF)/American Institute for Cancer Research (AICR)** and were advised to consume at least **1.2 g of protein/kg body weight/day and at least 25 g of protein per meal**. Control: Usual care during chemotherapy, which could include a referral to a dietitian when malnutrition was detected by the gynecological oncologist.	Relative dose intensity and progression-free survival
Carayol et al., 2019 (France) [39]	Randomized controlled trial	Randomized N = 143	Women aged 18–75 y; non-metastatic breast cancer; enrolled after curative surgery, planned for 6 cycles of adjuvant chemotherapy (epirubicin/cyclophosphamide/5-fluorouracil for 3 cycles every 3 weeks, followed by docetaxel for 3 cycles every 3 weeks), followed by 6 weeks of radiotherapy	Nutrition counseling and exercise	Trial duration: 26 weeks. Intervention: The Adapted Physical Activity and Diet (APAD) intervention consisted of exercise sessions planned thrice-weekly, nine hospital-based and face-to-face **nutritional therapeutic education sessions targeting body weight control and feeding behaviors according to the WCRF**. Specific advice was given to patients for the management of chemotherapy toxicities and side effects. Control: Usual care; no diet or exercise advice.	Cancer-related fatigue
Demark- Wahnefried et al., 2008 (United States) [40]	3-Arm randomized controlled trial	Randomized N = 90	Women with stage I–IIIA breast cancer scheduled for adjuvant chemotherapy (any type)	Calcium-rich diet ± exercise ± high fruit and vegetable, low fat	Trial duration: 6 months All groups: 14 contacts of 10–30 min each, once weekly for the first month and semiweekly for 5 months Intervention 1 (calcium-rich diet + exercise): Calcium-rich intervention + aerobic exercise ≥30 min per day, ≥3 times weekly and strength training exercises every other day. Intervention 2 (calcium-rich, low-fat, high-fruit-and-vegetable diet + exercise): Intervention 1 + goals of <20% energy from fat, ≥5 servings fruit + vegetables. Control (calcium-rich diet only): Written and verbal instruction on a calcium-rich diet (1200–1500 mg calcium/day).	Body composition (preservation of lean mass)
Djuric et al., 2011 (United States) [41]	Randomized controlled trial	Randomized N = 40	Females age ≥18 y with stage I–IIIA breast cancer scheduled for or within two weeks of starting chemotherapy (any type)	Nutrition counseling and exercise	Trial duration: 12 months. Intervention: Written educational materials and telephone counseling from a registered dietitian to achieve a **high**-**fruit-and-vegetable and low-fat** diet, based on an estimate of total energy expenditure (TEE). Control: “My Pyramid” plan from the United States Department of Agriculture and brochures from the American Cancer Society.	Feasibility
Jacot et al., 2020 (France) [42]	Randomized controlled trial	Randomized N = 360 Analyzed in a per-protocol analysis N = 321	Females ≥ 18 y with early-stage breast cancer, participants were enrolled after undergoing curative surgery and before 6 cycles of chemotherapy (six cycles of adjuvant chemotherapy [three cycles of epirubicin/cyclophosphamide/5-fluorouracil every 3 weeks, followed by three taxane-based cycles, either docetaxel every 3 weeks or paclitaxel weekly for 9 weeks]) followed by 6 weeks of radiotherapy.	Nutrition counseling and exercise	Trial duration: 26 weeks. Intervention: The Adapted Physical Activity Diet (APAD2) program: twice weekly exercise sessions and six nutrition sessions, which targeted body weight control and adherence to **WCRF recommendations**. Control: Usual care.	Cancer-related fatigue
Kenkhuis et al., 2024 (Netherlands) [60]	Same as Brouwer et al. [59]					Body composition, physical functioning, and fatigue
Maurer et al., 2022 (Germany) [43]	Randomized controlled trial	Randomized N = 15 Analyzed N = 11	Females aged ≥ 18 y with ovarian cancer, tubal cancer, or peritoneal cancer, scheduled for adjuvant or neoadjuvant chemotherapy (any type)	Nutrition counseling and exercise	Trial duration: 12 months. Intervention: Personalized exercise and nutrition counselling tailored to different phases of the patient’s treatment and recovery. The nutrition element included one-on-one sessions with a nutritionist every 3 weeks during chemotherapy, with a focus on consuming adequate **calories and protein**. After chemotherapy, monthly nutrition counselling was focused on the **Mediterranean diet**. Control: Usual care.	Feasibility (primary); secondary: quality of life, cancer-related fatigue, nutritional risk, physical activity, physical performance, body composition
Puklin et al., 2024 (United States) [61]	Randomized controlled trial	Randomized N = 173	Women with stage I–III breast cancer receiving chemotherapy (any type)	Nutrition counseling and exercise	Trial duration: Duration of chemotherapy (average 3.3 ± 1.2 months, range 8–24 weeks). Intervention: Goals to meet guidelines according to the **Healthy Eating Index-2015** [62] (≥5 fruits and/or vegetables/day, ≥25 g fiber/day, <30 g added sugar/day, ≤18 oz red meat/week, limited consumption of processed foods, ≤1 alcoholic drink/day) and **Physical Activity Guidelines** (≥150 min/week of moderate- to vigorous-intensity physical activity or 75 min/week of vigorous-intensity physical activity plus twice-weekly resistance training); a mean of 8 ± 3 counseling sessions were offered. Control: Usual care; access to a registered dietitian with referrals at the discretion of the treating oncologist.	Physical activity and diet quality
Raghunath et al., 2020 (India) [44]	Randomized controlled trial	Randomized N = 120	Males and females 18–65 y with stage II and III adenocarcinoma of the colon and prescribed chemotherapy (8 cycles with oxaliplatin and capecitabine for 14 days then 3 weeks off)	Naturopathy, yoga, and dietary counseling	Trial duration: 18 months. Intervention: The dietary intervention included a **schedule of foods such as vegetable juice, idlis, korralu, dalia, and milk malt with buttermilk**. Also, after the first cycle of chemotherapy, participants underwent a 7-day intensive phase of training in naturopathy and yoga. Control: Psycho-social counseling.	Hematological, biochemical, immunological, and psychological variables
Sanft et al., 2023 (United States) [45]	Same as Puklin et al. [61]					Relative dose intensity (measure of chemotherapy completion)
Stelten et al., 2022 (Netherlands) [46]	Randomized controlled trial	Randomized N = 28	Females aged ≥18 y with primary epithelial ovarian cancer scheduled to undergo first-line neoadjuvant chemotherapy (any type)	Nutrition counseling and exercise	Trial duration: Duration of chemotherapy plus follow-up 3 and 12 weeks after chemotherapy. Intervention: The dietary component involved individually tailored diets delivered via face-to-face sessions once every three weeks **to achieve/maintain nutritional status and dietary intake and to prevent weight loss**, guided by the WCRF/AICR (≥1.2 g protein/kg body weight/day evenly distributed throughout meals). The exercise component included two one-hour moderate to high-intensity resistance and aerobic exercise sessions per week by a physical therapist. Control: Usual care.	Body composition, physical function, and fatigue; though only qualitative data are reported in this paper.
***Nutrient-specific dietary patterns***						
Bille et al., 2018 (Denmark) [47] This manuscript also reports two pilot studies (n = 5 each) to select meals for the cross-over study. Details from the pilot studies are not described here.	Randomized cross-over controlled trial	Enrolled N = 62	Adults with hematological cancer being treated with chemotherapy (any type)	High energy density	Trial duration: 30-day control period followed by a 30-day intervention period. Intervention: Four **high-energy-density dishes** with protein content of at least 5 g/100 g each eaten once each: chili con carne, chicken in curry, curry soup, and pasta carbonara. Control: Habitual diet.	Acceptability of dishes, weight loss
Harvey et al., 2023 (United States) [6]	Same as Kleckner et al. [52]					Qualitative assessment of participants’ experiences
Gardner et al., 2008 (United States) [48]	Randomized controlled trial	Randomized N = 153	Males and females with acute myeloid leukemia receiving remission induction therapy	Neutropenic	Trial duration: Length of stay, 24–25 days. Intervention: No raw fruits or vegetables, only cooked. Control: A diet containing fresh fruit and fresh vegetables with encouraged consumption of at least one/day, with the fruits and vegetables washed with cold water for 30 s before being eaten.	Infection and death
IJmker-Hemink et al., 2021 (Netherlands) [49]	Randomized controlled trial	Randomized N = 20	Males and females aged ≥18 y with metastatic colorectal or gynecological malignancies starting with 3-weekly scheduled chemotherapy (any type)	Protein-rich home delivery	Intervention duration: 3 weeks (with follow-up for 3 months). Intervention: Home-delivered meal service of six protein-rich dishes per day based on the FoodforCare meal service. The meal service provided a morning shake, two lunch meals, a snack, dinner, and dessert for each day (average energy 1553 kcal/day, average protein 60.8 g/day) for 3 weeks. Participants received a leaflet with nutritional advice on protein-rich foods and recommendations for 1.2 g protein/kg body weight/day. Control: Usual care.	Quality of life
IJmker-Hemink et al., 2023 (Netherlands) [50]	Randomized controlled trial	Randomized N = 148	Males and females aged ≥18 y with cancer (any type) and receiving chemotherapy on a 2-, 3-, or 4-week cycle (any type)	Protein-rich home delivery	Same as IJmker-Hemink et al. [49]	Quality of life
Jalali et al., 2018 (Iran) [51]	Randomized controlled trial	Randomized N = 50	Males and females with acute myeloid leukemia undergoing chemotherapy (type not specified)	Mediterranean diet	Trial duration: 4 weeks. Intervention: Mediterranean diet with 47% carbohydrates, 15% protein, 38% fat (24% monounsaturated fatty acids mostly from olive oil), including 30 mL olive oil daily. Control: Neutropenic diet: cooked foods and vegetables, except for some raw fruits, including banana and orange; boiled water; pasteurized dairy; well cooked meat and eggs Both diets had a similar calorie count; the Mifflin formula was used to estimate energy needs.	Nutritional status
Kleckner et al., 2022 (United States) [52]	Randomized controlled trial	Randomized N = 33	Males and females aged ≥18 y with cancer (any type) with at least six weeks of chemotherapy remaining (any type)	Mediterranean diet	Trial duration: 8 weeks. Intervention: Participants received 12 frozen meals/week delivered to their homes once/week for four weeks and other grocery store items, a custom Mediterranean cookbook, and information describing the MedDiet. Participants had weekly phone-based check-ins with the study team and a one-on-one counseling session with a nutrition scientist during week 3. Control: Usual care.	Cancer-related fatigue
Sathiaraj et al., 2023 (India) [53]	Randomized controlled trial	Randomized N = 103	Females aged 18–65 years with nonmetastatic breast cancer and planning to have adjuvant chemotherapy (4 cycles of doxorubicin and cyclophosphamide or docetaxel and cyclophosphamide (1 cycle every 3 weeks)	Plant-based high protein	Trial duration: Duration of chemotherapy (about 12 weeks). Intervention: A **plant-based, high-protein diet** (1.2 g protein/kg body weight/day; 25–30% kcal from fat, 55–60% carbohydrates, 10–15% protein) aimed at weight management; advised no meat, included eggs and low-fat dairy, ≥5 servings fruits and vegetables, whole grain and millet, discouraged high-calorie fried food, soda; whey protein supplement (84 kcal, 10 g protein) once daily; educational materials; and weekly sessions/reviews. Control: Usual care, with access to on-demand nutrition appointments with the study team.	Cancer-related fatigue (primary), body composition (secondary)
Villarini et al., 2012 (Italy) [54]	Randomized controlled trial	Randomized N = 96	Women of any age, operated on for invasive, non-metastatic breast cancer, scheduled for adjuvant chemotherapy (any type)	Diet based on Mediterranean and macrobiotic recipes	Trial duration: Duration of chemotherapy. Intervention: Cooking classes and common meals at least twice per week in addition to the recommendations and kitchen course given to the control group; dietary recommendations based on **Mediterranean and macrobiotic recipes and on the avoidance of energy-dense foods**. Protein intake was reduced, as meat and cheese consumption was discouraged. Control: Recommendations for the prevention of cancer and a baseline kitchen course on how to reduce gastrointestinal side effects of chemotherapy.	Body weight
Zhang et al., 2023 (China) [55]	Randomized controlled trial	Enrolled N = 106	Patients with lung cancer undergoing chemotherapy (type not specified), body mass index 18.5–30 kg/m^2^, Brief Fatigue Inventory score of ≥4 points (range 0–10).	Anti-inflammatory diet	Trial duration: 3 months. Intervention: The **anti-inflammatory diet** goals: 2 servings of fruit, 5 servings of vegetables, 3 servings of whole grain cereals, 2 servings of nuts or seeds, 2 servings of olive oil or linseed oil, and 2 servings of probiotic low-fat dairy products per day; ≥3 servings of fish, ≤2 servings of red meat, and 5 servings of white meat per week; encouragement of certain spices and tea, discouragement of refined and processed foods; nutrition education; twice/week phone calls or face-to-face consultations. Control: Usual diet; 4 face-to-face visits for regular health education and recommendations based on dietary nutrition prescriptions for patients with cancer in China.	Cancer-related fatigue

Abbreviations: AICR, American Institute for Cancer Research; ESPEN, European Society of Parenteral and Enteral Nutrition; FIGO, Federation of Gynecology and Obstetrics; WCRF, World Cancer Research Foundation; WHO, World Health Organization.

**Table 2:** Feasibility, safety, and efficacy outcomes of nutrition studies conducted during chemotherapy.

Reference	Sample Characteristics	Feasibility (Completion, Attendance, Compliance)	Safety (Adverse Events, Side Effects)	Efficacy
***Nutritional counseling without exercise***				
Abdollahi et al., 2019 [19]	Analyzed N: 140 (73 intervention, 67 control) Sex: all female Mean age: 46.8 years Cancer type(s): breast	- 93.3% retention; 10/150 did not complete the study. - Compliance with the nutrition recommendations was >90% for the treatment group (details not described).	- None reported related to the intervention	- There were significant within-group decreases in the prevalence of **gastrointestinal side effects** in the intervention group in the third session of chemotherapy compared to the first session, including reflux disorder (*p* = 0.05), anorexia (*p* < 0.001), nausea (*p* = 0.002), constipation (*p* < 0.001), and diarrhea (*p* < 0.001); symptom prevalence was stable in the control group. - All 9 measured **gastrointestinal side effects** were lower in the intervention group vs. the control group at the fourth chemotherapy session, controlling for baseline presence and other potential confounding factors.
Baldwin et al., 2011 [20]	Analyzed N: 358 (90 diet, 86 supplement, 86 diet + supplement, 96 control) Sex: 256 males, 102 females Median age: 66.8 (range 24–88 years) Cancer type(s): 113 colorectal, 81 lung, 72 pancreas, 71 esophago-gastric, 10 liver and biliary, 7 unknown primary, 4 other	- 326/358 (91.1%) retention at week 6 (primary endpoint), 153/358 (42.7%) retention at week 52; data available for 98% because most lost-to-follow-ups were because of death. - Only 60/236 (25%) completed a food diary at baseline and 40 (17%) completed it at more than one time point (data loss due mostly to deteriorating health status and death); therefore, analysis was deemed not useful. - Supplement intake logs were returned by 62/150 (41%) participants; 31% took all prescribed supplements the first week and 19% took them all at week 6; 5 (8%) were unable to take any supplements the first week and 48% were unable to take any supplements by week 6	- No side effects reported that were attributed to the intervention	- **One-year survival** was 38.6% (95% confidence interval 33.3–43.9%), with no differences between groups (±dietary advice, ±supplement). - **Quality of life**, as measured using both the EORTC-C30 and the FAACT, were not different between the groups. - There were no between-group differences in **weight change**. - There were no between-group differences in **hand grip strength**.
Bourdel-Marchasson et al., 2014 [21]	Analyzed N: 336 (169 intervention, 167 control) Sex: 172 males, 164 females Mean age: 78.0 years Cancer type(s): colon (49), stomach (25), pancreas and cholangiocarcinoma (62), non-small cell lung (35), prostate (13), bladder (20), ovary (25), breast (28), lymphoma (50)	- 98.5% retention. - 877/990 (88.6%) dietitian visits were attended. - In both groups, energy intake increased between visit 1 and visit 2 (intervention: +328 kcal/day, *p* < 0.0001; control: +132 kcal/day, *p* = 0.02) but the difference was higher in the intervention vs. control group (*p* < 0.01). - At visit 2, 57 (40.4%) participants in the intervention group vs. 13 (13.5%) in the control group achieved the goal of 30 kcal/kg/d or more and 66 (46.8%) in the intervention group vs. 20 (20.8%) in the control group achieved the goal of 1.2 g protein/kg/d.	- None reported related to the intervention	- **One-year and two-year mortality** were similar in both groups. - **Clinical outcomes** of chemotherapy management, remission status, weight change, serum albumin, incidents of falls, hospitalization, and use of artificial nutrition were similar in both groups (*p* ≥ 0.19). - There were more **grade 3–4 infections** among control vs. intervention participants (*p* = 0.03).
Cao et al., 2024 (China) [56]	Analyzed N: 92 (45 intervention, 47 control) Sex: 77 males, 15 females Mean age: 61.1 years Cancer type(s): head and neck squamous cell carcinomas	- 92/94 (97.9%) completed the study. Two patients in the intervention group dropped out because chemotherapy was stopped.	- None reported related to the intervention	- Compared to the control group, patients in the intervention group had a significantly lower **incidence of nausea** (*p* = 0.021) and a significantly lower rate of **vomiting** (*p* = 0.035). - Regarding the patient-reported **severity of nausea and vomiting**, the intervention group showed better results compared to the control group (*p* = 0.014 and *p* = 0.029, respectively). - The scores of the nausea and vomiting domains of the **Functional Living Index-Emesis** in the intervention group were significantly higher than those in the control group (*p* < 0.001 and *p* = 0.018, respectively), indicating that patients in the intervention group perceived less impact of CINV on their daily lives.
Dai et al., 2022 [22]	Analyzed N: 61 (32 intervention, 29 control) Sex: 50 males, 11 females Mean age: 51.7 years Cancer type(s): nasopharynx (51), hypo-pharynx (3), tonsil (1), cervical lymph node (1), tongue (1), larynx (3), oropharynx (1)	- 84.7% retention; 11/72 (15.3%) did not complete the study. - The decrease in calorie intake was attenuated in the intervention group vs. control (not significant at week 2, *p* < 0.001 at weeks 4 and 6). - Protein intake increased in the intervention group and decreased in the control group (*p* < 0.001 between groups at all 3 time points).	- None reported related to the intervention	- **Body weight** was higher in the intervention vs. control group (*p* = 0.03). - **Karnofsky Performance Status** score was higher in the intervention vs. control groups across all time points (*p* = 0.01). - Decreases in **nutritional indexes** albumin, transferrin, and pre-albumin, and triceps skin folds were higher in the control vs. intervention group (*p* < 0.05). - **Anxiety and depression** were higher in the control vs. intervention group over all time points (*p* ≤ 0.01). - Participants in both groups suffered from **mucositis** to different degrees.
de Lima Bezerra et al., 2023 [23]	Analyzed N: 34 (19 intervention, 15 control) Sex: all females Mean age: 44–45 years Cancer type(s): breast	- 100% retention; no loss to follow-up.	- None reported related to the intervention	- There was less chemotherapy-induced **gastrointestinal chemotoxicity** in the intervention vs. control group for nausea (*p* < 0.001), vomiting (*p* = 0.048), and constipation (*p* = 0.033) but not diarrhea, anorexia, or mucositis. - The intervention group had a lower frequency of chemotherapy **dose-limiting toxicity** compared to the control group (15.8% vs. 26.7%, *p* = 0.672). - Changes in **body weight** were similar between groups. - There were no between-group differences in **disease-free survival**.
de Souza et al., 2021 [24]	Analyzed N: 34 (19 intervention, 15 control) Sex: all females Mean age: 44.3 years Cancer type(s): breast	- 30/34 (88.2%) completed the study.	- None reported related to the intervention	- **Global health status quality of life** increased slightly over time for both groups, with no between-group differences. - **Physical, emotional, cognitive, and social functioning** were stable over time for both groups. - **Role functioning** was maintained in the intervention group and decreased over time for the control group (*p* < 0.001 between groups). - **Anthropometrics** (body weight, BMI, and calf circumference) decreased slightly over time for both groups to a similar degree (*p* > 0.1 over time for both groups). - **Hand-grip strength** decreased significantly over time for the control group (*p* = 0.009), and the decrease was less in the intervention group (*p* = 0.125 over time). - **Nausea and vomiting** scores were higher in the intervention group vs. the control group (*p* < 0.001 group × time); appetite loss decreased over time for the intervention group and remained stable for the control group (but was higher at baseline in the intervention group); there were no other effects of group or group × time for other quality-of-life symptoms. - In regard to hematological and gastrointestinal toxicities, the intervention group had a lower incidence of **leukopenia** in the third cycle of chemotherapy (*p* = 0.034) and a lower amount of **abdominal pain** in the second cycle (*p* = 0.034); no other between-group differences.
Ford et al., 2024 (Canada) [57]	Analyzed N: 50 (25 intervention, 25 control) Sex: 30 males, 20 females Mean age: 57 years Cancer type(s): colorectal cancer	- 40/50 (80%) participants were retained (23/25 control and 17/25 intervention). - Both groups increased protein consumption from baseline to weeks 6 and 12, with those in the 2.0 g/kg group consuming more at week 6 and week 12. Being in the 2.0 g/kg/day group resulted in 0.3 g/kg/day greater protein intake compared with the 1.0 g/kg/day group (*p* = 0.019) across timepoints. The protein goal of 2.0 g/kg/day was achieved by 30% of the participants at week 6 and 35% at week 12.	- None reported related to the intervention	- Half of all participants (20/40) maintained or gained **muscle mass** by week 12. - Irrespective of group allocation, **percent change in muscle mass** from baseline trended toward a positive association with actual protein intake and suggested that an increase of 1.0 g/kg/day protein may result in a 1.6% increase in appendicular lean soft tissue index (*p* = 0.090). - An increase in protein consumption was associated with an increase in **physical function** as measured using the Short Physical Performance Battery (*p* = 0.014).
Keum et al., 2021 [25]	Analyzed in a per-protocol analysis: 33 (17 intervention, 16 control) Sex: 20 males, 13 females Mean age: 61.5 years (range 34–78 years) Cancer type(s): pancreatic ductal adenocarcinoma	- 34/40 (85.0%) were retained post-intervention at 12 weeks; 33/40 had evaluable data.	- None reported related to the intervention	- All the study participants showed a significant improvement in **nutritional status** (PG-SGA), with no between-group differences; those who used the Noom app more showed a significant improvement in PG-SGA. - The “above average” app users showed an increase in **body weight** and BMI compared to the “below average” users. - There was no statistically significant difference in **quality of life** between groups as measured using EORTC; there was an improvement over time for the intervention vs. control group as measured using the global health status and quality of life scale (*p* = 0.004). - **Skeletal muscle index** decreased for the non-app user and increased for the “above average” app user (*p* = 0.041). - A decrease in **tumor size** was more prominent in the “above average” user vs. the non-users (*p* = 0.17). - There were no between-group differences in **survival** metrics.
Lin et al., 2017 [26]	Analyzed N: 110 (55 intervention, 55 control) Sex: 70 males, 40 females Mean age: 52.6 years Cancer type(s): colorectal	- Retention not reported, implied 100% retention.	- None reported related to the intervention	- **Body weight** increased over time for the intervention group (*p* ≤ 0.001 at the 6th and 12th cycles) and not the control group (*p* > 0.24). - **Nutritional biomarkers** albumin and prealbumin increased over time for both groups, with higher increases in the intervention group. - There were no **treatment interruptions** from nutrition-related complications in the intervention group, but there were 8 cases in the control group.
Loprinzi et al., 1996 [27]	Analyzed N: 107 (54 intervention, 53 control) Sex: all females Median age: 43 (range 26–57) years Cancer type(s): breast	- Data available for 107/109 (98.2%) who entered the study.	- None reported related to the intervention	- The median **weight change** for the intervention group was +2.0 kg and for control group it was +3.5 kg 6 months after start of chemotherapy (not statistically significant). - More weight gain was seen in participants with higher Quetelet’s indexes (*p* < 0.01), those who had been on a diet in the previous 6 months (*p* < 0.025), those with higher scores on an introversion scale (*p* < 0.04), and those with higher scores on an obsessive/compulsive scale (*p* < 0.025). - There was no evidence of association between any weight change variable and appetite, nausea, or vomiting.
Najafi et al., 2019 [28]	Analyzed N: 137 (70 intervention, 75 control) Sex: all females Mean age: 46.9 ± 12.4 years intervention, 46.0 ± 8.8 years control Cancer type(s): breast	- Same as Abdollahi et al. [19] - The intervention group on average ate less fat and less protein over time and the control group did not, but comparisons to recommendations are not reported.	- None reported related to the intervention	- **Quality of life** was higher in the intervention vs. control groups and stable over time for both groups. - **Nausea**, as measured using three instruments, was less for the intervention group vs. the control group for all three chemotherapy sessions (*p* < 0.001). - Participants in the intervention group had higher levels of physical, role, emotional, and cognitive **functioning** across all three chemotherapy time points (*p* < 0.001 for all). - Participants in the intervention group had equal (dyspnea, insomnia, appetite loss, constipation, diarrhea) or less severity (fatigue, nausea and vomiting, pain) of all **symptoms** measured.
Ollenschläger et al., 1992 [29]	Analyzed N: 29 (16 intervention, 16 control) Sex: 16 males, 16 females Age range: 17–59 years Cancer type(s): acute leukemia	- 29/32 (90.6%) analyzed. - The mean dietary intake of those in the intervention group was 23.3 ± 11.4 kcal/kg ideal body weight during weeks of weight loss, 30.9 ± 13.1 during weeks with stable weight, and 39.3 ± 12.2 kcal (*p* < 0.0001). - Dietary intake was not compared to goals for the intervention group and was not reported for the control group.	- It is mentioned that toxicities occurred, but none were stated to be attributed to the intervention.	- There were no between-group differences in **septic episodes** or days with body temperature > 38.5 °C. - Participants in both study groups lost **body weight** up to the third or seventh study week. Among the participants treated with LAM-6 (*n* = 13), those in the intervention group showed more weeks with weight gain vs. those in the control group. There were no between-group differences for participants undergoing other types of treatment. At the end of induction therapy, only 5/16 participants in the intervention group showed a body weight <95% of their pre-study weight vs. 11/16 control participants. - Neither within-group nor between-group changes were reported for **well-being** or **quality of life**. Weight loss was positively correlated with weakness/malaise, energy intake was negatively correlated with weakness/fatigue, energy intake was negatively correlated with side effects of therapy, and quality of diet was negatively correlated with side effects of therapy (all *p* < 0.01).
Ovesen et al., 1993 [30]	Analyzed N: 117 (59 intervention, 58 control) Sex: 90 female, 27 male Mean age: 59 years (intervention), 58 years (control), range 22–80 years Cancer type(s): ovarian (*n* = 86), lung (*n* = 78), breast (*n* = 36)	- 105/137 (85.4%) analyzed. - Daily energy and protein intake did not change in the control group and increased significantly in the intervention group (~1 MJ [239 kcal] and 10 g of protein) at one month, which was sustained during the study period. - Dietary intake was close but did not meet goals for the intervention group, with a 1.5 ± 0.4 × basal energy expenditure and protein intake of 1.1 ± 0.4 g protein/kg vs. 1.3 ± 0.4 × basal energy expenditure and 0.9 ± 0.5 g protein/kg in the control group.	- No adverse events were reported.	- Both groups lost **body weight** in the first month of the study, but the intervention group gained more weight subsequently, but the difference did not meet statistical significance (*p* = 0.15). - In regard to **tumor response**, 65% were responders in the intervention vs. 69% in the control group at 3 months (*p* = 0.83). At 5 months, there were more responders in the intervention group (63%) vs. the control group (46%, *p* = 0.11). - There were no between-group effects on **quality of life**.
Regueme et al., 2021 [31]	Analyzed N: 155 Sex: 78 males, 77 females Mean age: 77.3 ± 4.8 years Cancer type(s): colon (43), lymphoma (34), lung (11), ovary (16), breast (9), stomach (9), pancreas (17), prostate (8), bladder (6), cholangiocarcinoma (1), unknown (1)	- Same study as Bourdel-Marchasson et al. [21]. - 155/283 (54.8%) analyzed (57 died before end of chemotherapy, 71 had missing data). - Dietary intervention increased total energy and protein intake (specifics not reported)	- None reported related to the intervention	- There were no between-group differences in **health-related quality of life;** physical functioning and fatigue decreased with time in some participants (23.9% and 36.9%, respectively). - Worsening of **comprehensive gerontological assessment items** was observed frequently for the Geriatric Depression Scale (51.6% of participants), timed up-and-go (50.0%), OLS (76.5%), activities of daily living (14.9%), and instrumental activities of daily living (34.8%); there were no between-group differences.
Sukaraphat et al., 2016 [32]	Analyzed N: 50 (25 intervention, 25 control) Sex: 34 male, 16 female Mean age: 61.3 years (intervention), 62.7 years (control) (range 45–81 years) Cancer type(s): lung (*n* = 35), cholangiocarcinoma (*n* = 15)	- 100% retention through post-intervention (12 weeks). - Energy intake was similar at baseline (mean 1415 kcal in the intervention group vs. 1510 in the control group, *p* = 0.48). Energy intake was greater in the intervention group than the control group at post-intervention (1832 ± 672 kcal vs. 1641 ± 343 kcal/day, *p* = 0.21) and 2 months post-intervention (1847 ± 443 kcal vs. 1615 ± 313 kcal/day, *p* = 0.06), but differences did not reach statistical significance.	- No adverse events were reported.	- The within-group change in **body weight** from pre- to post-chemotherapy was higher for the intervention group vs. the control group (2.3 ± 6.2% vs. −1.7 ± 6.2%, *p* = 0.03). Similar results were seen for BMI (difference in within-group changes *p* = 0.03). From pre-chemotherapy to 2 months post-chemotherapy, body weight increased 2.6 ± 6.4% in the intervention group and changed to −0.27 ± 7.1% in the control group (*p* = 0.19). - While scores were similar at baseline (*p* > 0.2), there was a lower PG-SGA score (better **nutritional status**) in the intervention vs. control group at post-intervention (*p* < 0.001) and 2 months post-intervention (*p* < 0.01). - **Quality of life** was similar at baseline and then greater in the intervention vs. control group at post-intervention (*p* = 0.01) but the significance did not hold 2 months post-intervention (*p* = 0.08). - There were no between-group differences in **serum albumin** or **total lymphocyte count** at baseline, post-intervention, or 2 months post-intervention. - There was a higher percentage of participants in the control group vs. the intervention group who **delayed chemotherapy** (48% vs. 36%), but the difference was not statistically significant (*p* = 0.57). - **Response to treatment** (stable/progression of disease, partial response) was similar between groups. - **Infection rates** were similar between groups (no statistics reported).
Tang et al., 2024 (China) [58]	Analyzed N: 60 (30 intervention, 30 control) Sex: 45 males, 15 females Mean age: 53.8 years Cancer type(s): nasopharyngeal carcinoma	- 100% retention. - Effective recovery rate of 100% for 60 questionnaires.	- None reported related to the intervention	- **NRS scores** were higher for the control group than the intervention group throughout the 24-week study (week 3, *p* = 0.014; week 10, *p* = 0.070; week 24, difference 0.33 ± 0.77, *p* = 0.023), indicating higher nutritional risk. - **Body weight** of the control group was lower than that of the intervention group throughout the study (mean difference of 3.9 kg at 12 weeks, *p* = 0.044). - **Quality of life** (as measured by SF-36 Scale) after 24 weeks showed higher scores (higher quality of life) for the intervention group vs. the control group for physiological function, somatic pain, general health, energy, and social function (*p* < 0.05).
van der Werf et al., 2020 [33]	Analyzed N: 72 (51 intervention, 54 control) Sex: 66 males, 41 females Mean age: 64 ± 13 years intervention, 66 ± 10 years control Cancer type(s): metastatic colorectal cancer	- 102/107 (95.3%) continued until T1 (after ~9 weeks of CAPOX(-B)/capecitabine(-B) or 12 weeks of FOLFOX(-B)). - 72/107 (67.2%) continued until T2 (mean 19 weeks). - The intervention group had a mean of 12.1 ± 4.6 dietetic consultations vs. 3.5 ± 2.5 in the control group.	- None reported related to the intervention	- 30% of the patients had a decrease in **skeletal muscle area** of ≥6.0 cm^2^ from T0 to T1, with no between-group differences in incidence (*p* = 0.47) or degree of change (*p* = 0.9 for both change from T0 to T1 and T1 to T2). - The intervention group increased in **body weight** from T0 to T1 1.7 kg (95% CI 0.0–3.3 kg) vs. −0.2 kg (−1.6–1.3 kg) in the control group. - There was no effect of group on **muscle density**, **hand grip strength**, **quality of life** global health score, physical functioning, or quality of life subscales. - There was no effect of group on the incidence of grade 3/4 toxicity or reduced treatment intensity. - Participants in the intervention group had significantly longer **progression-free survival** (*p* = 0.039) and **overall survival** (*p* = 0.046).
Wang et al., 2023 [34]	Analyzed N: 36 (18 intervention, 18 control) Sex: 28 males, 8 females Median age: 67.5 (range 54–78) years Cancer type(s): esophageal cancer (squamous cell carcinoma, 34; adenocarcinoma, 2)	- 36/53 (67.9%) completed the study.	- None reported related to the intervention	- In the intervention group, **albumin** decreased at first then recovered and was similar to baseline (*p* = 0.21), though it decreased in the control group over time (*p* = 0.01); similar trends were observed for **hemoglobin** (*p* = 0.09 for the intervention and *p* = 0.04 for the control group over time). - **C-reactive protein** levels increased during treatment, but then gradually decreased in both groups, though increases were more in the control group compared to baseline (*p* = 0.02). - The incidence of grade ≥ II lymphocytopenia was higher in the control group than the intervention group (33.3% vs. 61.1%, *p* = 0.03). - The incidence of **complications** including nausea, vomiting, leukopenia, radiation esophagitis, and myelosuppression were similar between groups. - There was no between-group difference in **remission status**. - The average **length of hospital stay** was 12 days less (47 vs. 35 days, *p* = 0.001), **and in-patient expenses** were CNY 20,504 less (*p* = 0.004) in the intervention group compared to the control group.
White et al., 2020 [35]	Analyzed N: 50 (24 intervention, 26 control) Completed the study: N = 40 (20 intervention, 20 control) Sex: 14 males, 36 females Mean age: 56.5 ± 13.9 intervention, 44.7 ± 14.4 control Cancer type(s): cervical (35), bladder (15)	- 100% remained at the study at 6 weeks and were therefore assessed for the primary outcome (treatment toxicity). - 48/50 (96%) had analyzable data at 12 weeks and 40/50 (80%) had analyzable data at 12 months. - It was possible to perform 57/72 (79%) of proposed intervention tests with no disruption of oncological management. - There was excellent compliance with questionnaires. - 151/179 (84%) of requested food diaries were completed.	- There were no adverse events attributable to the intervention.	- Participants in both groups experienced **fewer gastrointestinal symptoms**. At 6 weeks (primary endpoint), bowel scores were higher (worse) for the control vs. intervention group. - There were no grade 3–4 **bowel toxicities** in either group. - **Chemotherapy interruptions** due to bowel symptoms in 3 patients in the control group and 1 patient in the intervention group. - 17/24 (71%) participants in the intervention group **received the planned chemotherapy** vs. 16/26 (62%) in the control group. - There were no between-group differences in **anxiety**, **depression**, or **quality of life**. - Participant **ratings of satisfaction** were “very satisfied” or “extremely satisfied” in both groups.
Xie et al., 2017 [36]	Analyzed N: 144 Sex: 85 males, 59 females Mean age: 55.5 ± 11.4 years control, 55.3 ± 8.6 years intervention Cancer type(s): gastric cancer (stomach, 74; cardia, 31; antrum, 39).	- All randomized participants were included in analyses (100%). - The intervention group had a significantly higher calorie intake, ratio to recommended nutritional intake (RNI), and iron intake vs. the control group (all *p* < 0.05 for all) at the first chemotherapy session.	- None reported related to the intervention	- **Hemoglobin** was similar between groups at baseline and was higher in the intervention vs. control group for the 3rd–8th chemotherapy session (*p* < 0.05 for all time points). - **Serum total protein** was higher at the first chemotherapy session for the intervention vs. control group (*p* = 0.021) but similar for all other time points. - **Albumin** tended to be higher for the intervention vs. control and reached statistical significance at some time points. - Reductions in **body weight** were slightly attenuated in the intervention vs. control group, but differences did not reach statistical significance. - **Chemotherapy interference** was lower in the intervention group than the control group (completion of scheduled regimen: 73.61% vs. 55.56%, *p* = 0.024).
***Nutrition counseling plus exercise***				
Allen et al., 2022 [37]	Analyzed N: 48 (26 intervention, 28 control) Sex: 46 males, 8 females Mean age: 64 ± 8 years Cancer type(s): advanced esophagogastric cancer (adenocarcinoma, 48; squamous cell carcinoma, 6)	- 48/54 (92%) completed the study. - Attendance at the supervised exercise sessions was 76 ± 14% and adherence to the home exercises was 65 ± 27%; medical coaching adherence was 82 ± 20%.	- No intervention-related adverse events were reported to study staff.	- There was no between-group difference in **anaerobic threshold** (*p* = 0.57) or group×time interaction (*p* = 0.40). - Reductions in **peak VO_2_** were attenuated in the intervention group following neoadjuvant therapy (*p* = 0.022). - The intervention group had less **skeletal muscle loss** following neoadjuvant therapy (*p* = 0.049). There was no between-group difference in the incidence of sarcopenia (*p* = 0.404). - Decreases in **hand grip strength** were attenuated in the intervention vs. control group (*p* = 0.009). - Global health status **quality of life** was higher for the intervention group vs. the control group (*p* = 0.002 for group × time). - **Anxiety** and **depression** were lower in the intervention vs. control group preoperatively and postoperatively at 6 weeks and 6 months. - More intervention participants **completed chemotherapy** at the full dose (75% vs. 46%, *p* = 0.036). - **Pulmonary complications** occurred at a similar rate between groups (59% intervention vs. 78% controls, *p* = 0.079). - There were no between-group differences in median **length of hospital stay**, **30-day readmission**, or 3-year postoperative **cancer-related mortality**.
Basen-Engquist et al., 2020 [38]	Analyzed N: 37 (19 intervention, 18 control) at the end of the study Sex: all female Mean age: 49 years Cancer type(s): breast	- 73.7% retention (28/38). - 12/19 (63%) controls and 16/19 (84%) completed the assessment at T2 (end of chemotherapy). - Intervention participants attended an average of 80% of the in-person sessions and 43% of the telephone sessions. - 88% (*n* = 8) of participants reported at T2 that they were satisfied with the intervention, and 88% (*n* = 8) said they would recommend it to a friend.	- None reported related to the intervention	- **Body weight** fluctuated over the course of the study, and there was no effect of group (*p* = 0.083). - **Waist circumference** was significantly lower across the study in the intervention group compared to control (*p* = 0.019). - In regard to **quality of life**, the intervention group reported significantly greater improvements on the SF-36 vitality subscale than control participants at the end of chemotherapy (*p* = 0.005); there were no significant intervention effects for physical functioning, mental health, role physical, bodily pain, general health, social functioning, role emotional, or body image (*p* > 0.1).
Brouwer et al., 2024 (Netherlands) [59]	Analyzed N: 81 (40 intervention, 41 control) Sex: all female Average age: 59 years Cancer type(s): ovarian	- Feasibility described in Kenkhuis et al. [60].	- Safety reported in Kenkhuis et al. [60]	- The proportion of patients achieving a **relative** **dose intensity** ≥ 85% was 74.4% in the intervention group compared with 61.5% in the control group (odds ratio 2.04, 95% confidence interval 0.75 to 5.84). - The intervention effect on **progression free survival** was not statistically significant (hazard ratio 1.63, 95% confidence interval 0.82 to 3.23). At 18 months, the proportion of patients without disease progression was 73% in the intervention group and 51% in the control group (no statistics presented).
Carayol et al., 2019 [39]	Analyzed N: 135 Sex: All female Mean age: 52 years Cancer type(s): breast	- Overall retention was 135/143 (94.4%). - 72/72 (100%) of the intervention group and 63/71 (89%) completed the intervention period (26 weeks) and provided patient-reported outcomes. - Adherence to the Adapted Physical Activity and Diet (APAD) intervention was 67% for completed planned exercise sessions and 97% for completed scheduled diet sessions.	- No serious adverse events were attributed to exercise or diet intervention.	- **Fatigue** peaked during treatment for both groups. All dimensions of fatigue were significantly less for the intervention vs. control group at the end of chemotherapy and the end of radiotherapy (end of the intervention, except for general fatigue, *p* < 0.03). Fatigue was similar between groups at the 6-month follow-up but was higher in the control vs. intervention group at 1 year (*p* < 0.05). - **Quality of life** was lowest during treatment for both groups. Those in the intervention group had higher quality of life at all time points after baseline (*p* < 0.05). - **Anxiety** and **depression** were less for the intervention group vs. control at the mid- and post-intervention times points, but not at the 6-month or 1-year follow-up. - In regard to **muscle function**, lower-limb muscle endurance was higher in the intervention vs. control group at the end of radiotherapy; there were no between-group differences in power or force. - There were larger, favorable decreases in **body weight** and **body mass index** in the intervention vs. control group at the end of radiotherapy, with no differences in total muscle mass. - **Chemotherapy completion** rates were similar between groups (65.1% in the control group and 78.8% in the intervention group, *p* = 0.083).
Demark- Wahnefried et al., 2008 [40]	Analyzed N: 82 (27 calcium-rich (control), 26 calcium-rich plus exercise, 29 calcium-rich plus high fruits and vegetables, low fat + exercise) Sex: all female Mean age: 41.8 ± 5.6 Cancer type(s): breast	- 82/90 (91.1%) completed the study. - Attendance at the telephone counseling sessions was control > diet + exercise > exercise (*p* = 0.071). - More logs were submitted by the control group vs. the exercise and diet + exercise groups. - Calcium intake increased in all arms of the study (*p* = 0.02, baseline vs. 3 and 6 months); it was similar among all groups with 74–87% consuming ≥1200 mg per day. - The diet + exercise group had higher fruit and vegetable intakes and lower fat intakes at 3 months and 6 months vs. baseline; 16% in the diet + exercise group consumed ≤30% energy from fat and 39% reported intakes of ≥5 servings of fruit and vegetables per day. - The control diet received the highest feasibility scores, ranging from 1.3 to 1.5 (“very likely” to “likely”).	- No adverse events related to the intervention were reported.	- All measures of **adiposity** increased over time among all groups, with the exception of waist circumference, which decreased in the diet + exercise arm; at 6 months, the diet + exercise arm also had lower scores for percentage of body fat. - There were no within-group changes over time or between-group differences in **lean body mass**. - In regard to **bone density**, spine and hip t-scores were slightly lower at 6 months than at baseline for all arms, with no between-group differences. - In regard to **serologic biomarkers** (insulin, proinsulin, insulin-like growth factor-1, C-reactive protein, total cholesterol, high density lipoprotein cholesterol, low density lipoprotein cholesterol, sex hormone binding globulin, interleukin-1β, tumor necrosis factor-α receptor 2), there were no between-group differences. - Significant improvements in **quality of life** were observed over time among all groups, with no between-group differences. - No within-group differences over time or between-group differences were observed for **depression** or **anxiety**.
Djuric et al., 2011 [41]	Analyzed N: 30 Sex: all females Mean age: 52 Cancer type(s): breast	- 30/40 (75%) completed the study. - 70% of self-monitoring logs were submitted, only 2 participants submitted < 50% of requested logs. - The number of servings of fruits and vegetables per day increased in the intervention arm, and the mean reported intake at 12 months was just above the minimum intervention goal of 7 servings/day. - Serum total carotenoids increased in the intervention arm. - In the intervention arm, fruit and vegetable intakes at 12 months were 9.8 servings/day by screener vs. 7.3 servings/day by recalls, confirming over-estimates by the screener.	- None reported related to the intervention	- **Anthropometrics**: body weight, body fat, and waist circumference tended to decrease in the intervention group and increase in the control group. - **Well-being** improved in both study arms and the change reached statistical significance only in the intervention arm. - Small, favorable improvements in **insulin, glucose, and leptin** were noted in the intervention vs. control arm. - Participants in the intervention arm declared that the study was **helpful**, while those in the control arm said helpfulness was neutral.
Jacot et al., 2020 [42]	Analyzed N: 180 in the intent-to-treat analysis, 179 in the per-protocol analysis Sex: all female Mean age: 52.5 ± 9.9 years Cancer type(s): breast	- 89.2% analyzed. - 67/142 (47.2%) were at least 80% adherent. - The intervention increased physical activity (metabolic equivalents per week, *p* < 0.001) and engagement in moderate intensity recreational activities (*p* < 0.001) - Participants in the intervention vs. control arm more frequently met WHO dietary recommendations at T1 (end of chemotherapy, 81.4% vs. 61.9%, *p* < 0.001), 80.5% vs. 70.9%, *p* = 0.071), and T3 (86.6% vs. 68.1%, *p* = 0.002).	- None reported related to the intervention	- Compared to T0, general **fatigue** was similar between groups at T1 (end of chemotherapy, *p* = 0.274), T2 (end of radiation therapy, *p* = 0.157), and T3 (1 year after enrollment, *p* = 0.933). Physical and mental fatigue (from the Multidimensional Fatigue Inventory) and the symptom fatigue increased over time in both arms, but there were no between-group differences. In the per-protocol analysis, the intervention enhanced motivation (*p* = 0.003) and reduced mental fatigue (*p* = 0.069). - There were no between-group differences in **quality of life**. - **Emotional function** increased and **cognitive function** decreased over time. - A lower proportion of patients in the intervention arm (54.0%) vs. control arm (66.9%) had **depression** 1 year after enrollment. - There were no between-group differences in **body weight**.
Kenkhuis et al., 2024 (Netherlands) [60]	Analyzed N: 81 (40 intervention, 41 control) Sex: all female Average age: 59 years Cancer type(s): ovarian	- 64/81 (79%) retention. - In the intervention group, median attendance for the physiotherapist was 71.7%. - Among the 39 participants with available physical activity logs, 16 participants attended more than 75% of the prescribed exercise sessions. - The median exercise relative dose intensity ranged from 0 to 97.3% (mean 72.6%, SD 16.0). - Median attendance for dietary sessions was 80% (IQR: 50–100%), and among the 31 participants with data, 18 participants attended more than 75% of the prescribed dietary sessions. - There were no within-group or between-group differences in adherence to the WCRF/AICR lifestyle recommendations, the Dutch Healthy Diet Index, caloric intake, or protein intake.	- No adverse advents were recorded during the study.	- There were within-group increases in **fat-free mass** and **physical** **functioning** and decreases in **fatigue** over time, with no between-group differences. - No significant difference between the groups were found for FFM (β = −0.5 kg; 95% CI = −3.2; 2.1), physical functioning (β = 1.4; 95% CI = −5.4; 8.3), or fatigue (β = 0.7; 95% CI = −1.5; 2.8). - No between-group differences were found for **physical fitness**, health-related **quality of life**, **anxiety**, **depression**, **neuropathy**, or **sleep disturbances**.
Maurer et al., 2022 [43]	Analyzed N: 11 (5 intervention, 6 control) Sex: all females Median age: 58 (range 21–77 years) Cancer type(s): ovarian	- 11/15 (73.3%) participants completed the study. - Adherence to the nutrition intervention was 76.8% (phase I, 92.3%; phase II, 59.6%). - The intervention group increased their protein intake from 65.8 g/day at baseline to 107.9 g/day at the end of chemotherapy; this increase was greater than for the controls. - The intervention group increased calorie intake from 1860 kcal/day at baseline to 2389 kcal/day at the end of chemotherapy. - The intervention group increased the Mediterranean diet adherence score from a median of 7 at baseline to a median of 10 at week 52; scores were similar between groups.	No adverse events reported to the study team due to the intervention or in-person assessments.	- Participants in the intervention group increased their **hand grip strength** from a median of 22.0 kg to 24.8 kg, while the control group only increased from 21.8 kg to 22.4 kg. - **Health-related quality of life** increased from baseline to 52 weeks from 37.5 to 70.8 points in the intervention group and from 41.7 to 50.0 score points in the control group. - Both total and physical **fatigue** decreased from baseline to 52 weeks in both groups, and the decrease was larger in the intervention group.
Puklin et al., 2024 (United States) [61]	Analyzed N: 173 (87 intervention, 86 control) Sex: all female Mean Age: 53 ± 11 years Cancer type(s): breast	- A total of 149 women (86%) completed the 1-year (80 [92%] intervention; 69 [80%] control). - Among the 150 women with dietary assessments at baseline, 128 (85%) completed them at 1 year (72 [89%] intervention; 56 [81%] control). - On average, women in the intervention arm attended 15 (SD = 3) of 16 counseling sessions during the 1-year intervention. - Ninety percent of women attended at least 80% of the sessions (≥13 sessions) and 89% attended all 16 sessions.	- No adverse events were attributable to the intervention.	- At 1 year, 76% of women randomized to intervention adhered to the **physical activity** guideline compared with 24% of control women (*p* < 0.001). Over the year-long intervention, the intervention arm increased 136.1 min/week (*p* < 0.001) of physical activity more than the control arm. - Over the year-long intervention, those in the intervention group increased **diet quality** a mean of 4.9 points vs. 2.4 points in the control group (*p* = 0.08, not significant). In regard to individual **dietary components**, the intervention group consumed less added sugar than the control group (*p* = 0.02), though there were no other statistically significant differences. - Among women in the intervention arm, in the multivariable model for change in minutes/week of physical activity over 1 year, lower baseline fatigue was associated with greater improvement in minutes/week of physical activity (*p* = 0.04).
Raghunath et al., 2020 [44]	Analyzed N: 116 (58 intervention, 58 control) Sex: 58 males, 58 females Median age: 48 (range 18–65) years Cancer type(s): colorectal cancer	- 118/120 (98.3%) completed the study; two in the control arm developed recurrences and were removed from the analysis; two from the intervention group were also removed so the groups would be equal.	- None reported related to the intervention.	- **Hematological parameters** maintained similar patterns between groups over the course of treatment and post-treatment survivorship. - The intervention vs. control group exhibited lower levels of the **tumor marker carcinoembryonic antigen** after chemotherapy (*p* = 0.0003). - **Symptoms** of anxiety, depression, and distress, and symptom severity, increased for the control group and decreased for the intervention group over the course of the study (all *p* < 0.001). The Functional Living Index: Cancer (FLIC) decreased for the control group and increased for the intervention group (*p* < 0.0001).
Sanft et al., 2023 [45]	Analyzed N: 173 (87 intervention, 86 control) Sex: all female Mean age: 53 ± 11 years Cancer type(s): breast	- 173/173 (100%) analyzed. - There was 91% attendance at counseling sessions. - The intervention group increased servings of fruit and vegetables and dietary fiber, while the usual care group decreased intake over the course of chemotherapy/the study (*p* < 0.01, between-group difference).	- 55% in the intervention group and 63% in the control group experienced at least one nutrition impact symptom (*p* = 0.32), attributed to chemotherapy. - No adverse events were attributable to the intervention.	- Both groups experienced decreases in **body weight** (average 1 kg, *p* = 0.76 between groups). - **Relative dose intensity** was 92.9 ± 12.1% in the intervention group and 93.6 ± 11.1% in the control group (*p* = 0.69). - Among the 72 women who received neoadjuvant (vs. adjuvant) chemotherapy, those in the intervention arm were more likely to have a **partial complete response** (53% vs. 28%, *p* = 0.037).
Stelten et al., 2022 [46]	Analyzed N: 24 Sex: all females Mean age: 53.7 ± 10.6 years Cancer type(s): ovarian	- 20/24 (83.3%) completed the study. - Interviews were available for 14 participants in the intervention group and 10 in the control group. - Participants attended 33–133% (median 100%) of the prescribed dietary sessions.	- None reported related to the intervention.	- Patients appreciated guidance on exercise and nutrition and perceived benefits including improved physical fitness, quality of life, peer support, and recovery after surgery and/or chemotherapy cycles.
Zhang et al., 2023 [55]	Analyzed N: 97 (51 intervention, 46 control) Sex: 72 male, 26 female Mean age: 61.6 ± 9.6 years (intervention), 62.4 ± 8.1 (control) Cancer type(s): squamous cell carcinoma (*n* = 28), adenocarcinoma (*n* = 50), small cell or other lung cancer (*n* = 20)	- 102/106 (96.2%) were retained at post-intervention/post- chemotherapy (12 weeks). - Energy intake was similar between groups. Protein (g/day), fat (g/day), fiber, omega-3 fatty acids, and many micronutrients were significantly greater in the intervention vs. control groups (*p* < 0.001). Carbohydrates and cholesterol intakes were greater in the control vs. intervention groups (*p* < 0.001).	- No adverse events were reported.	- **Cancer-related fatigue** did not differ at baseline, week 4, or week 8 (all *p* > 0.05) but was significantly lower at week 12 (*p* < 0.01); fatigue decreased over time for both groups (*p* < 0.01) and there was a group×time interaction favoring the intervention group (*p* < 0.001). - For high-sensitivity C-reactive protein, a marker of **inflammation**, levels decreased over time for both groups from baseline through week 6 and week 8, then increased at week 12 for the control group only (*p* < 0.001 at week 12). The group×time interaction was significant (*p* < 0.001), favoring the intervention group. - **Nutritional status** tended to increase over time as measured with PG-SGA (*p* < 0.001 for time). The intervention group had lower PG-SGA levels (better nutritional status) at week 8 (*p* = 0.007) and week 12 (*p* = 0.03) but there was no group×time interaction. - **Serum albumin** levels tended to increase with time for the intervention group and remain stable for the control group (group × time interaction *p* = 0.048). - The intervention was associated with greater **quality of life**, especially emotional functioning, cognitive functioning, fatigue, and sleep disturbance (between-group differences *p* < 0.05 at week 12).
***Nutrient-specific dietary patterns***				
Bille et al., 2018 [47]	Analyzed N: 32 (with weight measurements) Sex: 18 males, 14 females Mean age: 52 ± 14 (with full registration of intake) 52.5 ± 16 (without full registration of intake) Cancer type(s): Acute and chronic leukemia and lymphoma, Hodgkin’s lymphoma, and non-Hodgkin’s lymphoma (specifics not described)	- 32/62 (51.6%) who signed the consent completed the study. - 18 patients had full registration of their intake and 14 patients had incomplete registrations. - All 32 participants who started the study completed it. - Compliance from weeks 1 to 4 (details not presented).	- None reported related to the intervention.	- Participants gained **body weight** (1.2 ± 1.9 kg, 2%) during the intervention period and lost weight (−2.8 ± 5.2 kg, 4%) in the control period. - Chicken in curry was the **dish most frequently consumed** in the home-cooked meals, and curry soup was the most frequently consumed prepared dish.
Harvey et al., 2023 [6]	Analyzed N: 29 (21 intervention, 8 control) Sex: 1 male, 28 female Mean age: 51.0 ± 15.1 years Cancer type(s): breast (27), other (2)	- Feasibility described in Kleckner et al. [52].	- Safety reported in Kleckner et al. [52].	- **Facilitators to adherence** included education, convenience, and empowerment. - **Barriers to adherence** included chemotherapy side effects, food preferences, and lifestyle changes.
Gardner et al., 2008 [48]	Analyzed N: 153 (78 intervention, 75 control) Sex: not reported Mean age: 64 years (range 17–88 years, intervention), 63 years (range 47–84 years, control) Cancer type(s): Acute myeloid leukemia (144), myelodysplastic syndrome (9)	- 100% of participants completed the study. - Compliance was noted with daily food diaries. All patients remained on the correct diet, although some in the control group did not eat a fresh fruit or vegetable every day.	- None reported related to the intervention.	- 29% in the intervention (cooked) group and 35% of patients in the control (raw) group developed a **major infection** (*p* = 0.60), and the rate was similar to incidence rates for people not randomized in the study. - **Complete response rates** were similar between groups (56% in the intervention, 61% in the control, 64% in the non-randomized). - 53% in the intervention and 57% control had an **antecedent hematologic disorder**. - The number of days spent with **neutrophil counts** < 500/µL and <100 µL were similar between groups (*p* = 0.77 and *p* = 0.53, respectively). - 28 patients in both the cooked and raw groups **lost weight** (a median of 5 lb in each group) over the course of the study. - Incidence of grade 3 or 4 **mucositis** or **diarrhea** was similar between groups. - Fever of unknown origin occurred in 51% of the cooked group and 36% of the raw group.
IJmker-Hemink et al., 2021 [49]	Analyzed N: 13 (7 control, 9 intervention) Sex: 6 males, 14 females at baseline Mean age: 59 ± 14 years control, 63 ± 10 years intervention Cancer type(s): gastrointestinal (8), breast (4), gynecological (8)	- 13/20 (65%) completed the study after 35% dropouts. - 90% of participants completed their symptom diaries.	- None reported related to the intervention.	- Of 17 participants, 88% (n = 15) graded the **level of burden** (from 0 to 10) that they experienced by participating in the pilot study; the median level of burden was 2, ranging from 0 to 3. - The main **reason for participating** was to help future patients.
IJmker-Hemink et al., 2023 [50]	Analyzed N: 116 (69 control, 67 intervention) Sex: 38 male, 98 female Mean age: 59.0 ± 13.9 years control, 62.9 ± 9.5 years intervention Cancer type(s): breast (40), colorectal (39), gynecological (28), other (19)	- 136/148 (92%) completed randomization, 116/148 (78.3%) completed the intervention, and 96/148 (64.9%) completed the 3-month follow-up assessments. - Energy intake was 21% higher in the intervention group after 3 weeks of the intervention (*p* < 0.05). In the intervention group, 31% met protein requirements before the intervention and 47% met them after, compared to 28% and 33% of controls at the same time points.	- None reported related to the intervention.	- After the intervention, **quality of life** was 5.0 points higher for the intervention vs. control group (*p* = 0.122); the score was similar between groups at follow-up. - There were no within-group or between-group differences in **handgrip strength** or **nutritional status** (PG-SGA). - The Short **Physical Performance** battery increased in the intervention group from pre- to post-intervention (*p* < 0.05). - Emotional functioning was higher in the intervention vs. control group after the intervention (*p* < 0.05). Pain and tiredness were lower in the intervention vs. control group after the intervention. There were no other between-group or within-group differences in **functional** or **symptom scales**.
Jalali et al., 2018 [51]	Analyzed N: 50 (25 intervention, 25 control) Sex: 29 males, 21 females Mean age: 41.2 ± 14.2 years intervention, 40.8 ± 12.7 years control Cancer type(s): acute myeloid leukemia	- No dropouts reported, implying 100% retention.	- None reported related to the intervention.	- **Serum albumin** increased significantly more in the intervention group vs. the control group over the course of the study (*p* = 0.01); albumin decreased with time in the control group (*p* = 0.03). - **Protein intake** increased significantly in the intervention group from pre- to post-study (*p* = 0.001), but not in the control group (*p* = 0.2), though intake was similar between groups at the post-study time point (*p* = 0.26). - **Body weight** increased in the intervention group over time (*p* = 0.049) and decreased slightly in the control group (*p* = 0.13); weight was similar post-intervention.
Kleckner et al., 2022 [52]	Analyzed N: 33 (23 intervention, 10 control) Sex: 2 males, 31 females Mean age: 51.0 ± 14.6 y Cancer type(s): breast (*n* = 30), other (3)	- 100% of the participants completed the study. At week 4, diet adherence scores increased from baseline by 2.7 ± 3.2 points for the intervention group and 0.7 ± 2.4 points for the control group.	- None reported related to the intervention.	- The intervention had small–moderate beneficial effect on **cancer-related fatigue** at week 4 (effect size = 0.31; 95% CI = −0.44–1.06) and week 8 (effect size = 0.25; 95% CI = −0.50−0.99). - There were small between-group differences in **quality of life**, favoring the intervention group (effect size = 0.26; 95% CI = 0.55−0.94). - There were no within-group changes or between-group differences in **body weight**, **lipid profiles**, **homocysteine**, or **fructosamine**. - There were no between-group differences in **mitochondrial function** (basal respiration, maximal respiration, or spare capacity).
Sathiaraj et al., 2023 [53]	Analyzed N: 103 (52 intervention, 51 control) Sex: all females Mean age: 52 ± 9.7 y Cancer type(s): breast	- Retention not stated, implied to be 100%. - 50/52 (96%) of those in the intervention group adhered to the personalized nutritional counseling and 47/52 (90%) adhered to the whey protein supplement intake.	- None reported related to the intervention.	- **Fatigue** decreased over time in the intervention group and increased in the control group (*p* < 0.001). - The intervention group reported greater reductions in **total energy and** increases in **protein and fiber intake** (*p* < 0.001 for all three at post-intervention). - **Body mass index** decreased by 0.7 ± 0.8 kg/m^2^ in the intervention group, and the decrease was 0.4 ± 1.3 kg/m^2^ in the control group (*p* = 0.015). - **Fat mass** decreased in the intervention group (*p* < 0.001), with no within-group change in the control group. - **Muscle mass** decreased in the control group and was maintained in the intervention group (*p* < 0.05). Handgrip strength decreased slightly over time in the control group and was maintained in the intervention group, but differences were not statistically significant (*p* = 0.238). - **Chemotherapy completion rate**: relative dose intensity was similar between groups (91% having >90% in both groups).
Villarini et al., 2012 [54]	Analyzed N: 94 (47 intervention, 47 control) Sex: all females Mean age: 52.7 ± 10.8 years intervention, 48.4 ± 9.4 years control Cancer type(s): breast	- 94/96 (97.9%) completed the study. - As for the 24 h recalls collected between chemotherapy administration, the women in the intervention group showed a significantly higher frequency of the consumption of whole grain cereals (2.2 vs. 0.8 times per day) and legumes (0.5 vs. 0.3 times per day), and a significantly lower frequency of the consumption of sugar (0.4 vs. 0.9 times per day), refined cereal products (1.1 vs. 2.1 times per day), and dairy products (0.5 vs. 0.9 times per day) compared with the control group (*p* < 0.01 for all).	- None reported related to the intervention.	- The intervention group showed a significant reduction in **body weight** (2.9 kg on average) vs. 0.1 kg in the control group (*p* = 0.0001 for difference in pre-post changes). - Women in the intervention group lost 2.29 kg of **fat mass** vs. 0.69 kg in the control group. (*p* = 0.03). Women in the intervention group lost 0.67 kg **fat-free mass** vs. 0.06 kg in the control group (*p* = 0.01). - In regard to **hematological parameters**, red blood cells, white blood cells, platelets, and hemoglobin levels significantly decreased between baseline and the end of treatment in both intervention and control groups, with no between-group differences.

Abbreviations: AICR, American Institute for Cancer Research; BMI, body mass index; CAPOX, capecitabine and oxaliplatin; CI, confidence interval; CINV, chemotherapy-induced nausea and vomiting; CNY, Chinese yuan renminbi; EORTC, European Organization for Research and Treatment of Cancer; FAACT, Functional Assessment of Anorexia/Cachexia Therapy; FOLFOX, folinic acid, fluorouracil, and oxaliplatin; IQR, interquartile range; PG-SGA, Patient-Generated Subjective Global Assessment; QLQ-C30, Quality of Life Core Questionnaire-30; RNI, recommended nutritional intake; SD, standard deviation; VO_2_, volume of oxygen; WCRF, World Cancer Research Foundation; WHO, World Health Organization.

## Data Availability

All data in this project were obtained from publicly available publications.

## Supplementary Materials

The following supporting information can be downloaded at: https://www.mdpi.com/article/10.3390/curroncol32010003/s1, Table S1: Search terms; Figure S1: Risk of bias assessment.
