# Supplementary material for: Nutritional Counseling During Chemotherapy Treatment: A Systematic Review of Feasibility, Safety, and Efficacy
Source: Curr Oncol. 2024 Dec 24;32(1):3. doi: 10.3390/curroncol32010003 (PMC11764068; doi:10.3390/curroncol32010003)
Supplement: Supplementary file 1 [file curroncol-32-00003-s001.zip › curroncol-3323161-supplementary.pdf]

Supplemental Information

Table S1. Search terms.

**Database**	**Type of Search**	**Search Query**
EMBASE	Broad search terms, Emtree, Boolean operators	#1. (diet* OR nutri*) AND during AND cancer AND chemo*:ab,ti<br>#2. #1 AND [adult]/lim AND 'article'/it
SCOPUS	Advanced search, Boolean operators	TITLE-ABS-KEY (diet* AND during AND cancer AND chemo*) AND (LIMIT-TO (DOCTYPE , "ar"))
Cochrane Library	Advanced search, MeSH terms, Boolean operators	(diet* OR nutrition*):ti,ab,kw AND (during):ti,ab,kw AND (cancer):ti,ab,kw AND (chemo*):ti,ab,kw

Intention-to-treat

Unique ID	D1	D2	D3	D4	D5	Overall
Abdollahi2019	+	+	+	+	+	+
Allen2021	+	+	+	+	+	+
Baldwin2011	+	+	!	+	!	!
Basen-Engquist2020	!	+	+	+	+	+
Bille2018	!	!	+	+	+	!
Bourdel-Marchasson2014	+	+	+	+	+	+
Brouwer2024	+	+	+	+	+	+
Cao2024	+	+	+	+	+	+
Carayol2019	+	+	+	+	!	+
Dai2022	!	+	+	+	!	!
deLimaBezerra2023	+	+	+	+	!	!
Demark-Wahnefried2008	+	+	+	+	!	!
deSouza2021	+	+	+	+	!	+
Djuric2012	+	+	+	+	+	+
Ford2024	+	+	+	+	+	+
Gardner2008	!	+	+	+	!	!
Harvie2022	+	+	+	+	+	+
IJmker-Hemink2021	+	+	+	+	!	+
Jacot2020	+	+	+	+	!	!
Jalali2018	+	+	+	+	!	!
Kenkhuis2024	+	+	+	+	+	+
Keum2021	+	+	+	+	!	!
Kleckner2022	+	+	+	+	+	+
Loprinzi1996	+	+	+	+	!	!
Maurer2022	+	+	+	+	!	!
Najafi2018	+	+	+	+	+	+
Ollenschlager1992	!	+	+	+	!	!
Omar2022	+	+	+	+	!	!
Ovesen1993	+	+	!	+	!	!
Puklin2024	+	+	+	+	+	+
Raghunath2020	+	+	+	+	!	!
Regueme2020	+	+	!	!	+	!
Sanft2023	+	+	+	+	+	+
Sathiaraj2022	+	+	+	+	!	!

+ Low risk
! Some concerns
- High risk

D1 Randomisation process
D2 Deviations from the intended interventions
D3 Missing outcome data
D4 Measurement of the outcome
D5 Selection of the reported result

Stelten2022	+	+	+	+	+	+
Sukaraphat2016	!	+	+	+	!	!
van der Werf2020	+	+	+	+	+	+
Villarini2012	+	+	+	+	!	!
Wang2023	+	+	!	+	!	!
White2020	+	+	+	+	!	!
Xie2017	-	+	+	+	!	-
Zhang2023	!	+	+	+	!	!

Figure S1. Risk of bias assessment.
